# Immunomodulatory Protective Effects of Rb9 Cyclic-Peptide in a Metastatic Melanoma Setting and the Involvement of Dendritic Cells

**DOI:** 10.3389/fimmu.2019.03122

**Published:** 2020-01-15

**Authors:** Fabrício C. Machado, Natália Girola, Vera S. C. Maia, Patrícia C. Bergami-Santos, Alice S. Morais, Ricardo A. Azevedo, Carlos R. Figueiredo, José A. M. Barbuto, Luiz R. Travassos

**Affiliations:** ^1^Recepta Bio, São Paulo, Brazil; ^2^Experimental Oncology Unit, Department of Microbiology, Immunology and Parasitology, Federal University of São Paulo, São Paulo, Brazil; ^3^Tumor Immunology Laboratory, Department of Immunology, Biomedical Sciences Institute, University of São Paulo, São Paulo, Brazil; ^4^MediCity, University of Turku, Turku, Finland

**Keywords:** metastatic melanoma, cyclic-peptide, cytokines, MIF-CD74, dendritic cells, macrophage differentiation, lymphocyte proliferation

## Abstract

The cyclic VHCDR3-derived peptide (Rb9) from RebMab200 antibody, directed to a NaPi2B phosphate-transport protein, displayed anti-metastatic melanoma activity at 50–300 μg intraperitoneally injected in syngeneic mice. Immune deficient mice failed to respond to the peptide protective effect. Rb9 induced increased CD8+ T and low Foxp3+ T cell infiltration in lung metastases and high IFN-γ and low TGF-β in lymphoid organs. The peptide co-localized with F-actin and a nuclear site in dendritic cells and specifically bound to MIF and CD74 in a dot-blot setting. Murine bone-marrow dendritic cells preincubated with Rb9 for 6 h were treated with MIF for short time periods. The modulated responses showed stimulation of CD74 and inhibition of pPI3K, pERK, and pNF-κB as compared to MIF alone. Rb9 in a melanoma-conditioned medium, stimulated the M1 type conversion in bone marrow-macrophages. Functional aspects of Rb9 *in vivo* were studied in therapeutic and prophylactic protocols using a melanoma metastatic model. In both protocols Rb9 exhibited a marked anti-melanoma protection. Human dendritic cells were also investigated showing increased expression of surface markers in response to Rb9 incubation. Rb9 either stimulated or slightly inhibited moDCs submitted to inhibitory (TGF-β and IL-10) or activating (LPS) conditions, respectively. Lymphocyte proliferation was obtained with moDCs stimulated by Rb9 and tumor cell lysate. In moDCs from cancer patients Rb9 exerted immunomodulatory activities depending on their functional status. The peptide may inhibit over-stimulated cells, stimulate poorly activated and suppressed cells, or cause instead, little phenotypic and functional alterations. Recently, the interaction MIF-CD74 has been associated to PD-L1 expression and IFN-γ, suggesting a target for melanoma treatment. The effects described for Rb9 and the protection against metastatic melanoma may suggest the possibility of a peptide reagent that could be relevant when associated to modern immunotherapeutic procedures.

## Introduction

Cancer is a leading cause of human death with high incidence in low, middle and high-income countries (1, 2). Malignant neoplasms derive from normal tissue with abnormal and excessive cellular growth, caused by genetic mutations and epigenetic modifications, leading to tumor masses formation. The progressive accumulation of cellular changes may give to the transformed cells the ability to invade adjacent tissues and spread to distant sites through the lymphatic and blood circulatory systems, forming metastases. Immune suppression can be induced at this stage and the untreated or treatment resistant cancers can be fatal (3, 4).

Monoclonal antibodies (mAbs) immunotherapy and chemotherapeutic agents may target tumor antigens and be effective because of their specificity and efficacy with acceptable side effects (5–7). The ability to modulate immune responses has become an important strategy in antibody cancer therapies (8–10). Recently, mAbs targeting immune checkpoints have been used to treat various solid tumors and lymphomas, but the low response rate and adverse events indicate the need for predictive biomarkers to improve the applicability of anti-PD-1/PD-L1 and anti-CTLA-4 agents (11).

Apart from mAbs specifically targeting tumor antigens, receptors and co-signaling molecules of the immune system, bioactive peptides from a number of sources have been studied with various specificities and affinities for microorganisms and eukaryotic cells (12, 13). For more than a decade, peptides derived from immunoglobulin (Ig) internal sequences have been shown to display differential anti-infective and anti-tumor activities *in vitro* and *in vivo* (14, 15). Different peptides can also be immunomodulatory by activating signaling pathways, stimulate, or regulate the expression of maturation markers on dendritic cells, stimulate antigen presentation, cytokine production, and lymphocyte interaction, phenotypes that will define the ultimate immune response (16, 17). High rates of resistance and relapse in anticancer treatment stimulate the search for additional agents, able to modulate dendritic cells and effector or regulatory T lymphocytes, memory T and B lymphocytes, which could improve the anti-infective or anti-tumor effectiveness of the immune response (18, 19).

In addition to the beneficial effects of delaying or arresting growth of certain types of neoplasms, current anticancer drugs may otherwise cause impairment of antibody synthesis, auto-immunity, and several side effects that altogether stimulate the research for new agents able to control the growth of neoplastic cells (20, 21). The present work focus on the anti-tumor effect of an immunologically bioactive synthetic peptide, Rb9, derived from the complementarity determining region-3 (CDR3) of V_H_ from a humanized monoclonal antibody (RebmAb 200) to NaPi2b transporter (22). The anti-tumor protective effects of Rb9 against metastatic melanoma, which depends on a healthy immune response and immune-modulatory activation of murine or human dendritic cells, and the possible molecular mechanism of this response were further investigated in the present work as an important step to the development of new anticancer drugs.

## Materials and Methods

### Mice

Six- to eight-week-old male C57Bl/6, Balb/c, or NOD/Scid/IL-2R-γ^null^ mice were acquired from the Center for Development of Experimental Models (CEDEME) at Federal University of São Paulo (UNIFESP), Brazil. Mice were housed in ventilated racks in specific pathogen free conditions (SPF) for at least 1 week with *ad libitum* access to water and food in a light:dark cycle of 12 h each, before experimentation.

### Ethics Statement

Animal experiments were carried out in accordance with the recommendations of the National Council for the Control of Animal Experimentation (CONCEA, Brazil) and approved by the Ethics Committee of Federal University of São Paulo, registered with the number CEUA 3521121217. All methods were performed in accordance with relevant guidelines and regulations approved by UNIFESP. Standard clinical symptoms that indicate deteriorating health conditions requiring euthanasia before the end of the experiment were followed.

The study with human donor and patient cells was carried out in accordance with the recommendations of Research Ethical Committee (CEP, Brazil) from the Faculty of Medicine, São Paulo University (FMUSP, Brazil). The protocol was approved ref. number 457.616 on Nov 13, 2013.

### Peptides

Peptide Rb9 sequence is derived from V_H_ CDR3 of a humanized monoclonal antibody (RebmAb 200), which reacts with an epitope in the sodium-phosphate transporter NaPi2b (23–26). Synthetic Rb9, however, displays bio-reactivity independent of mAb specificity (22). The synthesis of Rb9 and other peptides was carried out by Peptide 2.0 (Chantilly, VA). Modifications included amidation at the C terminus (NH_2_) and cyclization (C-C, disulfide bridge). A solution of Rb9 lyophilized material was prepared diluting the peptide in milli-Q water at 10–15 mM and further dilution for use, in RPMI 1640 medium or PBS. Linear sequences are shown below:

**Table:** 

**Peptide**	**Sequence**	**Modification**
Rb9	CARGETARATFAYWGQGC	C-term NH_2_, (C-C)
Scr-Rb9	TFAYWRAACACGQGRTEG	C-term NH_2_
Rb10A1	AARGETARATFAYWGQG	C-term NH_2_

Both scrambled (Scr-Rb9) and Rb10A1 peptides were derived from the linear form Rb10, analogous to RB9, without C-terminal QGC residues. Rb10A1 is a negative control for *in vitro* experiments replacing Cys in the N-terminal by Ala.

### Tumor Cell Lines and Cell Cultures

B16F10-Nex2 subline of murine melanoma B16F10 (27), isolated and maintained at the Experimental Oncology Unit (UNONEX) of Federal University of São Paulo (UNIFESP) was deposited in the “Banco de Células do Rio de Janeiro” (BCRJ), no. 0342. The original B16F10 cell line was obtained from the Ludwig Institute for Cancer Research (LICR), São Paulo Branch. Additional syngeneic tumor cells were kindly donated by Dr. G. Mazzolini from Gene therapy Laboratory, School of Medicine, Austral University; Buenos Aires, Argentina. The murine pancreatic carcinoma Panc02 cells, syngeneic in H-2^b^ C57Bl/6 and the colon-rectal carcinoma CT26 cells syngeneic in H-2^d^ Balb/c mice were tested *in vivo* via subcutaneous (s.c.) grafting (Supplementary Figure 1). Tumor cell lines and primary isolated cells were cultivated at 37°C, under humid atmosphere and 5% CO_2_, in R10, which consisted in RPMI-1640 medium supplemented with 10 mM *N*-2-hydroxyethylpiperazine-*N*-2 ethane sulfonic acid (HEPES), 24 mM sodium bicarbonate, 40 mg/L gentamicin, 100 mg/L streptomycin, pH 7.2 and 10% fetal bovine serum (FBS). For some experiments, D10 medium was used (DMEM supplemented with 10% of FBS, 100 mM sodium pyruvate, 1x MEM-nonessential amino acids, 200 mM glutamine, 1x MEM vitamin solution, 0.05 M β-mercaptoethanol, 10,000 U penicillin and 10 mg/mL streptomycin). The protocol to obtain fresh isolated tissue cells is detailed below.

B16F10 melanoma cells were cultured in R10 until 70% confluence when the medium was harvested and fresh medium was added (v/v) to obtain a B16-conditioned medium (B16.CM). After three subculturings in the conditioned medium, B16.CM was collected, filtered, and used in functional assays.

### Induced Melanoma Metastatic Model

In the lung metastatic-melanoma colonization model, 2–4 x 10^5^ viable B16F10-Nex2 cells resuspended in 100 μL of serum-free RPMI medium, were intravenously (i.v.) injected in C57BL/6 or NOD/SCID/IL-2Rγ^null^ mice. Each inoculated mouse received 50–300 μg Rb9, Rb10A1 (a negative *in vitro* control), or Scr-Rb9 (a scrambled control version of Rb9) peptide diluted in 100 μL PBS via intraperitoneal (i.p.) administration or subcutaneous, in the interscapular area (s.c.). The peptide treatment occurred for 5–6 alternate days, starting 1 or 2 days after tumor cells injection. The control group (Veh) received a mock inoculation of PBS with the same volume. Anti-PD-1 (BioCell, InVivoPlus, clone J43) was used to treat mice at 6.25 mg/kg in 5 alternate days. Fifteen to twenty-two days later, mice were euthanized and their lungs were harvested. Macroscopic melanotic nodules were counted and images from the entire lung were captured with a stereomicroscope Nikon SMZ745T (magnification, 4x) coupled with Nikon Digital Sight DS-Fi2 and DS-U3 (Nikon Corporation, JA). The images were also used to measure the lung area with melanotic nodules. This quantification was obtained and measured by outlining the surface area covered by the black metastatic nodules in relation to the total organ area using Fiji/Image J version 1.52e software.

### Pancreatic and Colorectal Subcutaneous Solid Tumor Model

In the subcutaneous tumor model, 5 × 10^5^ syngeneic tumor cells resuspended in 100 μL of PBS were injected s.c. in the right flank of mice. C57Bl/6 mice were inoculated with Panc02 cells and Balb/c mice were inoculated with CT26 cells. Anti-tumor treatments with Rb9 or controls were performed as described before. Tumor longitudinal diameter (D) and transverse diameter (d) were measured by caliper rule every 2 days until the tumor volume reached 3,000 mm^3^. Animals were euthanized when the allowed volume was reached. The tumor volume was calculated by the formula V = D.d.2 × 0.52.

### Lung Tissue Digestion and Flow Cytometry Analysis

Lungs with metastatic nodules were excised from metastatic B16F10-Nex2 melanoma-bearing mice and pooled for tissue digestion, using 40 U/mL DNAse (Sigma Aldrich), 125 U/mL Collagenase type IV (Sigma Aldrich), 100 U/mL Hyaluronidase (Sigma Aldrich), and 0.025 mg/mL Liberase™ (Sigma Aldrich) in PBS supplemented with 0.5% BSA. Pooled lung samples from each group were shaken for 2 h at 37°C and the digestion was interrupted by adding 1 mM EDTA. The liquid from digested samples was fully homogenized by softly pipetting and was then filtered through a 40-μM cell strainer (BD Falcon). Digested samples were kept at 4°C during centrifugation at 1,000 *g* for 5 min and incubated for 5 min in ACK buffer (NH_4_Cl 150 mM, KHCO_3_ 1 mM, and Na_2_-EDTA 0.1 mM) for red cell lysis before another centrifugation for washing. Viable tissue cells from digested lung were counted in a Neubauer chamber using Trypan blue (Gibco™) and 1 × 10^6^ cells were collected to analyze the following cell markers by flow cytometry.

The following antibodies were used in two combinations to analyze the collected tissue cells: 1 anti-CD3 (MACS, Miltenyi Biotec), anti-CD4 (MACS, Miltenyi Biotec), anti-CD8 (MACS, Miltenyi Biotec) and 2) anti-CD4 (MACS, Miltenyi Biotec), anti-CD25 (MACS, Miltenyi Biotec), anti-NKG2D (Invitrogen), and anti-Foxp3 (MACS, Miltenyi Biotec). In general, cells were stained in PBS, 0.5% BSA with 1 mM EDTA for 30 min using the appropriate antibody-fluorophore conjugate. Cells were washed in cold PBS with BSA and suspended in fixation buffer (PBS, BSA with 2% paraformaldehyde) before flow cytometry. For Foxp3 staining, cells were fixed and permeabilized using solutions from the MACS, Miltenyi Biotec kit before staining. Compensated multiparameter analysis was performed on a BD FACSCanto II analyzer (BD Biosciences, USA) with FlowJo X software (Tree Star Inc., USA).

### ELISA for Cytokine Secretion

Mice challenged with B16F10-Nex2 tumor cells, received 300 μg i.p. of Rb9 or Rb10A1 and were euthanized after 17 days and their spleen and lymph nodes were collected, macerated, filtered through a cell strainer and washed 1x in PBS 1x. Splenocytes obtained after incubation in ACK hemolysis buffer were plated at 10^6^ cells per well in 6-well plates with R10 medium. On the same day, splenocytes were further incubated for 72 h with or without tumor cell lysate obtained from freezing and thawing B16F10-Nex2 cells, adding the equivalent of 10^5^ cells/well in triplicate. Lymph node cells were sorted for CD11c+ before incubation for 24 h with or without tumor cell lysate and supernatant collection. After incubation period, the supernatants were collected from both tissue cell culture and used to cytokine quantification by ELISA, following the manufacturer recommendations (BD Biosciences, USA). Briefly, a 96-well opaque plate (Nunc, Roskilde, Denmark) was coated overnight at 4°C with IL-6, IL-10, IL-12, IFN-γ, TNF, and TGF-β capture antibody, following the manufacturer recommendations of respective kits. The plates were blocked for 2 h at room temperature and washed with 0.05% Tween 20-PBS (T-PBS) before incubation for 2 h with previously collected supernatants and respective recombinant proteins. After incubation, the plate was washed with T-PBS. Biotin-conjugated detection antibody were incubated for 1 h, with streptavidin-peroxidase to signal amplification. The evaluation of absorbance was performed by OPD (Sigma Aldrich) in a multiplate reader (SpectraMax M2e, Molecular Devices, USA) at 450 nm.

### Immunohistochemistry

Tumor-bearing mice, received 200 μg i.p. of Rb9 or Scr-Rb9 and were euthanized after 15 days. Their lungs were surgically excised and cryopreserved in Tissue-Tek compound (Sakura Europe) at −80°C. Standard 5 μm sections were obtained from paraffin embedded lungs cuts laid on cleaned glass slides to immunohistochemistry preparation. Briefly, fixed sections were deparaffinized, rehydrated with graded xylene-ethanol series and endogenous peroxidases were inhibited by two washes of 3% hydrogen peroxide in methanol. Non-specific antigen-antibody reaction was blocked before staining with monoclonal anti-CD4 (Spring Biosciences Corp.) and monoclonal anti-granzyme B (Dako) antibodies. The immunohistochemical analysis was carried out using the peroxidase-conjugated Avidin-biotin complex and 3,3-diaminobenzidine (DAB) peroxidase substrate following standard procedures. The antigen-antibody reaction was visualized as a brown precipitate and stained tissue was counterstained with hematoxylin for 3 min. The slides were then rehydrated and mounted for observation under light microscopy. Photomicrographs were taken of each slide and the signal intensity was measured on images of several lung nodules using color deconvolution with Fiji/Image J version 1.52e software.

### Bone Marrow Derived Dendritic Cells and Macrophages

Bone marrow-derived dendritic cells (bmDCs) were obtained from C57Bl/6 mice as previously described (28–30). Briefly, mice femura and tibiae were stripped out of muscles and tendons and bone ends were cut to flushed out the bone marrow using a 26-gauge needle and syringe with R10 medium. Cell clusters were dissociated by passing through a cell strainer and red cell lysis were carried out using ACK buffer. The bone marrow cells were cultured in 100-mm tissue culture dish with 10 ml of R10 medium supplemented with GM-CSF (50 ng/ml,) and IL-4 (50 ng/ml). Fresh medium (3 ml) with GM-CSF (100 ng/ml) and IL-4 (100 ng/ml) was added every 3 days. On day 7, non-adherent cells were collected and pooled with adherent cells, which were harvested by PBS with 2 mM EDTA. Bone marrow-derived macrophages (bmMΦs), were extracted from femura and tibiae incubated with M-CSF-1 (10 ng/mL) in complete D10 medium for 6 days.

### Immunofluorescence

Murine bmDCs were plated on glass slides (Tekdon Inc.) inside 24 well plates at a concentration of 2.5 × 10^4^ cells in 60 μL for 1 h. After adhesion, 300 μL R10 medium was added and the cells were incubated overnight. On the next day, bmDCs were incubated with 500 μM of Rb9 conjugated with biotin for 1, 3, 8, and 24 h. A negative control consisted of cells without peptide added. The culture medium was removed and the slides were washed 3x with PBS before fixation in PBS containing 3.7% of formaldehyde for 20 min. Cells were then washed 3x with PBS, permeabilized with PBS containing 0.01% Triton X-100 for 5 min and then incubated with PBS containing 0.25% BSA for 1 h at room temperature in order to block non-specific sites. Finally, cells were stained for 1 h at room temperature in the dark using the same blocking buffer supplement with phalloidin-rhodamine for actin cytoskeleton, 10 μg/mL 4',6-diamidino-2-phenylindole (DAPI) for nucleic acid and anti-biotin-FITC to localize the Rb9 peptide. Glass slides were washed 5x with PBS and prepared as glass covers using Vectashield (VectorLabs) and sealed with nail polish before visualization in TCS SP5 II Tandem Scanner (Leica) confocal microscope with a 63 × NA 1.40 PlanApo oil immersion objective.

### Chemiluminescent Dot-Blotting

Rb9 and Rb10A1 peptides were diluted at 10 μg/10 μL in milli-Q water and applied on nitrocellulose membranes. Recombinant MIF and CD74 (Abcam, UK) were applied onto the nitrocellulose membranes at 50 nM and incubated overnight at 4°C. After washing, membranes were incubated with anti-MIF or anti-CD74 for 1 h at 37°C followed by several washes and anti-rabbit and anti-mouse IgG-HRP antibody incubation for 1 h at 37°C. Immunoreactivity was determined using the Luminata™ Forte solution (Millipore, Billerica, MA) and images were acquired by Uvitec Cambridge (Cambridge, UK). Some other peptides were also evaluated with negative reactivity (data not shown). This protocol was adapted from previous studies (31, 32).

### Immunoblotting

Cultured bmDCs were treated with 200 μM Rb9 for 6 h and then incubated with 1 μg/mL recombinant MIF (rMIF) for 2, 5, 10, and 20 min before cells were lysed, centrifuged at 1,000 *g* for 5 min and washed 1x in TBS (50 mM Tris-HCl pH 7.5, 150 mM NaCl) before add Laemmli buffer (62.5 mM Tris-HCl, pH 6.8 at 25°C, 2% w/v SDS, 10% glycerol, 50 mM DTT, 0.01% w/v bromophenol blue) and heat denaturation at 95°C for 5 min. Electrophoresis in polyacrylamide gels containing SDS and transfer to PVDF Immobilon P membrane (Millipore, Darmstadt, Germany) were carried out by standard procedures. The membranes were stained with 0.5 % Ponceau S in 3% acetic acid and eventually cut separating proteins with mass above and below 50 kDa. All membranes were blocked with TBS (10 mM Tris-HCl, 150 mM NaCl, pH 8) containing 0.05% Tween 20 (TBS-T) and 5% BSA overnight. Membranes were incubated for 3 h with anti-Akt, anti-pAKT (S473), anti-ERK1/2, anti-pERK1/2, anti-NF-κB pr65, anti-pNF-κB pr65 (S536), anti-PI3K pr85, anti-pPI3K pr85 (T458), anti-IkBα, anti-pIkBα (S32), anti-CD74, and anti-GAPDH, antibodies, purchased from Cell Signaling Technology (Beverly, MA) except for anti-GAPDH, acquired from Sigma-Aldrich (St. Louis, MO). The primary antibody was washed 3x in TBS-T for 10 min each and incubated for 1 h with anti-rabbit or anti-mouse IgG peroxidase-conjugated antibody (Thermo Fisher Scientific) diluted 1:20,000 in TBS. Membranes were washed 3x in TBS-T for 10 min. Immunoreactivity was determined using the Luminata™ Forte solution (Millipore, Billerica, MA) and images were acquired by Uvitec Cambridge (Cambridge, UK). Densitometry of bands was obtained using ImageJ software. pProtein/Protein ratios as in **Figures 6B–E** were normalized in relation to the ratios of unstimulated control cells (plotted = 1). For instance, sample 3 of pAkt: 9171.50/11358.86 = 0.8074 and CTR 1895.08/11373.45 = 0.1666; 0.8074/0.1666 = 4.84; or pAkt/Akt = 4.83/0.999 with CTR 1.00/1.00.

### Adoptive Cell Transfer Treatment

Two different protocols of adoptive cell transfer (ACT) treatment were performed. To the therapeutic protocol, murine bmDCs obtained as described above were stimulated with 50 μg/mL of Rb9 or Rb10A1 for 24 h. Mice previously challenged with B16F10-Nex2 were inoculated with 5 × 10^5^ bmDCs via i.p. per animal on the eighth day of tumor injection. The protective effect induced by Rb9 was evaluated after 15 days of tumor challenge by counting pulmonary melanotic nodules. In the prophylactic protocol of ACT, bmDCs were stimulated by Rb9 or Rb10A1, primed or not with tumor cell lysate (Lys, 1:10 v/v cell lysate from 5 × 10^4^ tumor cells) for 24 h, before s.c. inoculation in naïve mice. Each naïve mouse received two inoculations of primed bmDCs, on the 2nd and 7th days before B16F10-Nex2 challenge. The protective effect induced by Rb9 was also evaluated after 15 days of tumor challenge by counting pulmonary melanotic nodules.

### Human PBMC-Derived Dendritic Cell Obtention and Lymphocyte Proliferation

Blood samples from healthy donor and cancer patients (*n* = 22) were collected and peripheral blood mononuclear cells (PBMCs) were isolated by centrifugation over Ficoll-Paque Plus (GE Healthcare). After a 2 h-incubation in plastic 6-well plates, non-adherent cells were removed from culture and adherent cells (monocytes) were cultivated for 7 days in RPMI-1640 culture medium supplemented with 10% FCS, antibiotic-antimycotic agents (Gibco, Grand Island, NY, USA), in the presence of GM-CSF (50 ng/ml—Peprotech, Mexico) and IL-4 (50 ng/ml—Peprotech). At day 5 of culture TNF-α (50 ng/ml; Peprotech, Mexico) was added for monocyte derived DC maturation (mDCs). After maturation, mDCs received treatment with Rb9 or tumor cell lysate (Lys) before co-culture with allogeneic lymphocytes (DC:Ly = 1:30) to evaluate their ability to induce lymphocyte proliferation. Phytohemagglutinin (PHA) was also used as a control. The proliferation was measured by carboxyfluorescein succinimidyl ester (CFSE—Molecular Probes) dilution and activation was measured by correlation between CD4/CD25 and Foxp3 expression.

### Flow Cytometry Analysis of Dendritic and Lymphocytic Cells

BmDCs from mice were stained for CD11c, CD11b, and CD74 after treatment with 50 μg/mL Rb9 for 48 h and 200 ng/mL LPS for 24 h, and bmMΦs were stained for F4/80, MHC-II, CD86, PD-L1, and CD206 in PBS, 0.5% BSA for 30 min using the different antibody-fluorophore conjugates from MACS, Miltenyi Biotec. Cells were washed in cold PBS with BSA and suspended in fixation buffer (PBS, BSA with 2% paraformaldehyde) before flow cytometry. Compensated multiparameter analysis was performed on a BD FACSCanto II analyzer (BD Biosciences, USA) with FlowJo X software (Tree Star Inc., USA).

Human cells were analyzed similarly to mice cells, but the staining was performed in BSA-PBS without EDTA. To flow cytometry analysis was used antibodies against CD11c, CD14, CD80, CD83, CD86, HLA-ABC, HLA-DR, CCR7, and PD-L1, conjugated with different fluorochromes, besideslive/dead labeling (Molecular Probes, Oregon, USA). Acquisition was performed in a FACSCanto II analyzer (BD Biosciences), analyzed with the FlowJo Software X.10.07r2 (Tree Star). The frequency of FoxP3+ human cells was analyzed using the e-Bioscience Foxp3/Transcription Factor Staining Buffer Set (Affymetrix, e-Bioscience, USA) as described in the manufacturer's protocol. Before intracellular staining, the cells were labeled with fluorescence labeled anti-CD4, anti-CD8, and anti-CD25 (BD Biosciences).

### Statistics Analysis

The software GraphPad Prism version 7.0 (San Diego, CA) was used in all tests for significance analyses. Student, Welch, or Mann–Whitney *t*-test compared statistical differences between groups. One-Way ANOVA with Bonferroni correction; repeated measures ANOVA with Dunnett's correction, and two-way ANOVA were also applied. A difference in survival time was measured by Log-rank with Mantel-Cox test. *P*-values are indicated as ^*^p < 0.05, ^**^p < 0.01, and ^***^p < 0.001 indicating the significant difference. The X^2^ (chi-squared) test was used to determine the statistical significance of different frequencies from flow cytometer data.

## Results

### Anti-metastatic and Anti-tumor Activity of Rb9 Peptide

**Figure 1:**
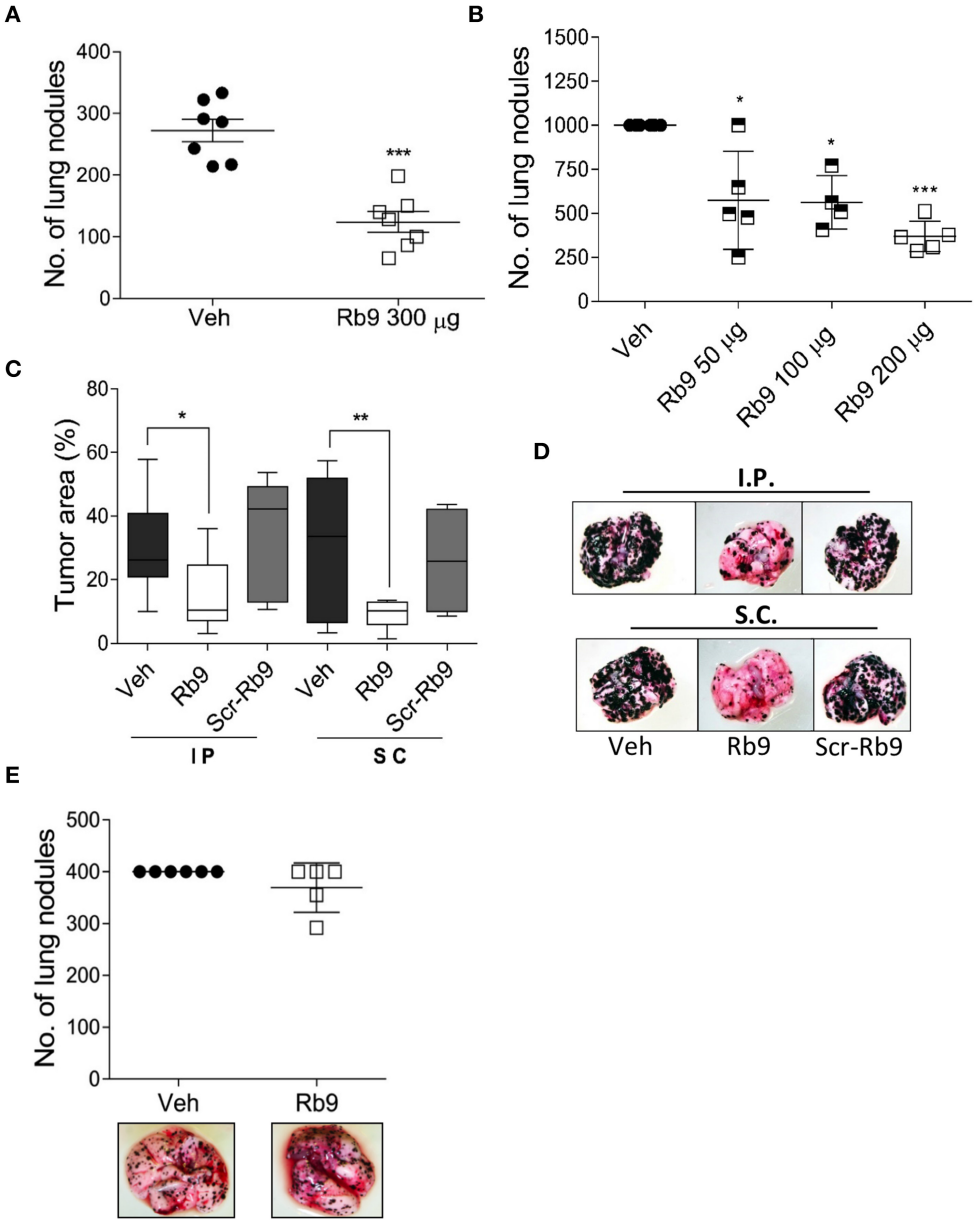
Rb9 peptide inhibits *in vivo* development of melanoma metastasis in immunocompetent mice. **(A)** Rb9 intraperitoneal (i.p) administration of 300 μg per animal for 5 alternate days reduce the number of B16F10-Nex2 lung metastatic nodules as compared to vehicle (Veh, PBS); **(B)** Different doses of i.p. Rb9 administration, 15 days of tumor challenge. ^*^p < 0.05 and ^***^p < 0.001 calculated using Student's or Welch's *t*-test, respectively. Vehicle (≥10^3^ counts); **(C)** Melanotic area on the lung surface, after 15-days tumor cell-challenge. Subcutaneously (s.c.) or intraperitoneally (i.p.) Rb9-treated mice, scrambled peptide (Scr-Rb9). Median, 25 and 75% quartiles, ± max and min values. ^*^p < 0.05 and ^**^p < 0.01. One-Way ANOVA with Bonferroni correction; **(D)** Representative panel of lungs after i.p. or s.c. Rb9 administration; **(E)** Immuno-compromised mice (NOD/Scid/IL-2Rγ^null^) are not capable to arrest melanoma metastasis development after treatment with 300 μg of Rb9.

B16F10-Nex2 cells were intravenously inoculated in C57BL/6 mice to generate a syngeneic model of pulmonary metastatic melanoma (33, 34). The anti-tumor activity of Rb9 was quantified in tumor cell challenged mice treated with 300 μg of Rb9 peptide per animal via i.p., starting 1 day after tumor cell inoculation followed by 5 doses in alternate days (Figure 1A). The number of pulmonary tumor nodules reduced when mice were injected with Rb9 in relation to control groups (Veh), which received only PBS, indicating the protective role of the peptide against melanoma lung colonization (metastasis). Similar results were obtained using different numbers of melanoma cells and different peptide concentrations, ranging from 50 to 200 μg of Rb9 per animal (Figure 1B). However, no protection was observed at 10 or 30 μg (not shown). Due to the variation of lung nodule size and the merge of individual nodules, the area of melanotic nodules over the lung surface was also measured (Figure 1C). In this experiment, mice received 6 inoculations of PBS, as a negative control group, or 200 μg of Rb9 per animal and equal amounts of Scr-Rb9 peptide i.p. and s.c. The tumor melanotic metastatic areas decreased significantly only after Rb9 treatment using both i.p. and s.c. administration routes. The lowest decrease was obtained with Rb9 s.c. treatment, as also observed in Figure 1D, which shows representative lung images from this experiment.

Although i.p. Rb9 treatment was efficient in the control of metastatic melanoma progression in immunocompetent mice, this peptide failed to promote a protective activity in immune-deficient mice (NOD/SCID/IL-2Rγ^null^), i.v. challenged with B16F10-Nex2 cells (Figure 1E). In fact, the *in vivo* anti-tumor activity of Rb9 seems to be tightly depended on healthy, non-immune compromised syngeneic mice. To further verify an immune response induced by Rb9 inoculation in tumor-bearing mice, sera collected from mice that received Rb9 *via* i.p. or s.c were applied into melanoma cells adhered onto plastic plates blocked or not with BSA. Increased reactivity to plated B16F10-Nex2 cells was observed for both sera. The serum from mice inoculated s.c. with Rb9 was even more reactive with melanoma cells than the serum from animals inoculated i.p. with the peptide (not shown).

In addition to melanoma cells, Rb9 protective activity was also tested against syngeneic CT26 colon and Panc02 pancreatic cancer cells grafted subcutaneously in C57Bl/6 mice (Supplementary Figure 1). The peptide was administered in 3 μg/μL/animal, via i.p. for 6 times in alternate days, starting 1 day after tumor cell grafting. In both cases the subcutaneous tumor progression was delayed with full survival of Panc02 after 40 days treatment (Supplementary Figure 1C) and none of colon cancer challenged mice after 60 days (not shown).

### Rb9 Modulates Cell Recruitment and Immune Activity in the Lung and Lymphoid Organs

**Figure 2:**
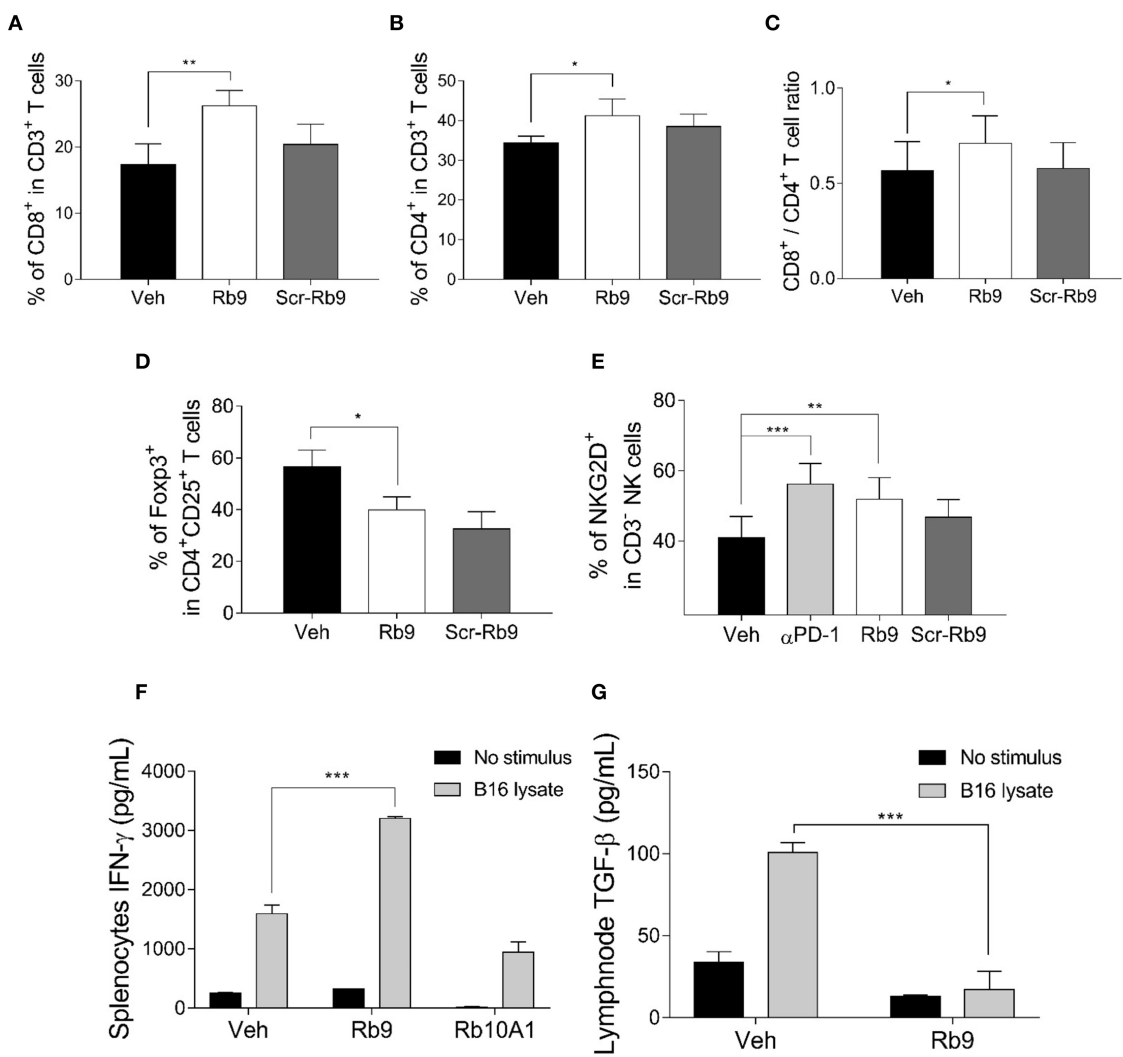
Lung lymphocyte recruitment in Rb9-treated mice and specific cytokine expression in splenocytes and CD11+ lymph node cells. **(A)** Percent CD8+ T cells inside lungs collected after 15 days from mice receiving 200 μg of s.c. Rb9 or Scr-Rb9 for 6 alternate days starting on day 2 after challenge with B16F10-Nex2 cells; **(B)** Percent CD4+ T cells inside lungs as in **(A)**; **(C)** Ratio of CD8+ T and CD4+ T lung infiltrates significantly increased in s.c. Rb9-treated mice. Values are means ± SEM of the previous experiments; **(D)** Percent CD4+, CD25+, Foxp3+ T cells inside lungs and **(E)** Percent CD3–NKG2D+ natural killer cells inside lungs as in **(A,B)**. Values are ± SEM of three experiments with 4–5 pooled lungs; ^**^p < 0.01 and ^*^p < 0.05 with repeated measures (RM)-ANOVA and Dunnett's post-test compared to Veh. **(F)** Splenocytes from 17-day melanoma cell challenged mice and Rb9 or Rb10A1 i.p. treatment, for 5 alternate days, after challenge on the 1st day. The splenocytes cell culture supernatant was used to measure IFN-γ secretion after 72-h stimulus with B16F10-Nex2 lysate; **(G)** CD11c+ cells from cervical and axillary lymph nodes were used to measure TGF-β reduced expression on cells after 24 h with tumor lysate stimulus. Graphs from **(F)** to **(G)** represent means ± SD of triplicate experiments quantified by ELISA using standard controls. ^***^p < 0.001.

As indicated above, the immune system is involved in the protective effect of Rb9 against metastatic melanoma. To further characterize this effect, the lung microenvironment and immune response in peptide-treated tumor-bearing mice were examined. The T lymphocyte population recruited in the lung microenvironment showed cells expressing CD3+, CD4+, CD8+, CD25+, and Foxp3+. We also evaluated NK cells by the expression of NKG2D marker in CD3- cells (Figure 2). After gating lymphocytes and measuring the cell population expressing both T-CD3+ and T-CD8+ an expressive increase of this population was observed in samples of s.c. Rb9-treated mice compared to Veh or Scr-Rb9 control groups (Figure 2A). Only a small increase of T-CD4+/T-CD3+ cells was observed in the same Rb9-treated samples (Figure 2B). Since the protective effect of Rb9 could be due to the modulation of lymphocyte recruitment and activation, the ratio of CD8+ T cells to CD4+ T cells was compared in lungs collected from different groups of treatment and it was increased only in the Rb9-treated group (Figure 2C). Inversely to T-CD4+ and T-CD8+ increased populations, the regulatory T lymphocytes expressing CD4+, CD25+, and Foxp3+ T lymphocytes were less expressed in Rb9-treated tissue samples (Figure 2D). Finally, NK cells defined with a similar size and granularity as T cells but without CD3 expression were evaluated using the activation marker NKG2D (Figure 2E) showing an increased recruitment of these cells similarly to the pulmonary recruitment of CD3-NKG2D+ cell after treatment with 6.25 mg/kg of murine anti-PD-1 therapy.

**Figure 3:**
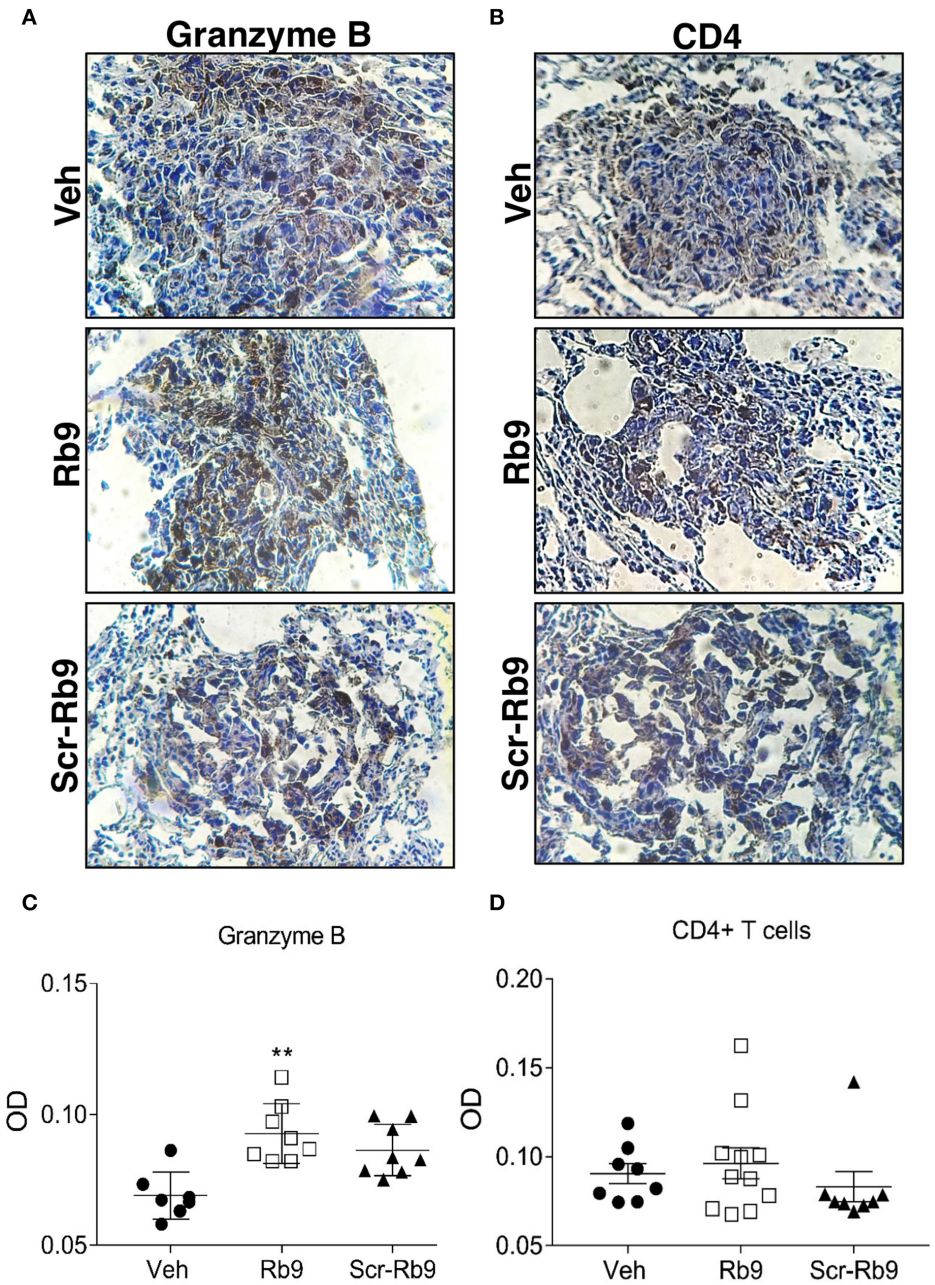
Immunohistochemistry of increased granzyme B secretion around lung metastatic nodules in Rb9-treated mice. **(A,B)** Panels show lung tissue including B16F10-Nex2 metastatic nodules in Rb9-treated mice. Immunohistochemistry staining with 3,3-diaminobenzidine for granzyme B **(A)** and CD4+ T cells **(B)**. Counterstaining: hematoxylin, in blue. Magnification: x400. **(C,D)** Graphs represent individual nodule areas, means and ± SEM of optical densities (OD) calculated after color deconvolution on ImageJ software for at least 8 nodules on each experimental group. ^**^p < 0.01 Mann–Whitney *t*-test as compared to Veh.

Immunohistochemistry of metastatic nodules in Rb9-treated mice showed that T-CD8+ and NK cells could be responsible for the secretion of granzyme B in the lung tumor tissue microenvironment from Rb9-treated mice (Figures 3A,C). The expression of T-CD4+ in metastatic nodules was only a little increased in Rb9-treated mice (Figures 3B,D).

Splenocytes isolated from Rb9-treated melanoma-bearing mice and thereafter stimulated with melanoma lysate were able to produce increased levels of IFN-γ as compared to splenocytes isolated from tumor-bearing mice treated with Veh or Rb10A1 control groups (Figure 2F). Axillary and cervical lymph node cells secreted low TGF-β under similar conditions (Figure 2G). Rb9 did not modify the melanoma lysate response in relation to IL-12, TNF, and IL-10 secretion but significantly reduced IL-6 (Supplementary Figure 2).

Altogether, these results suggest an immunomodulatory activity of Rb9 leading to anti-tumor response mediated by cytotoxic T-CD8+, NK cells, and IFN-γ with low TGF-β and Treg lymphocytes.

### Rb9 Interacts With Murine Bone-Marrow Dendritic Cells (bmDCs)

**Figure 4:**
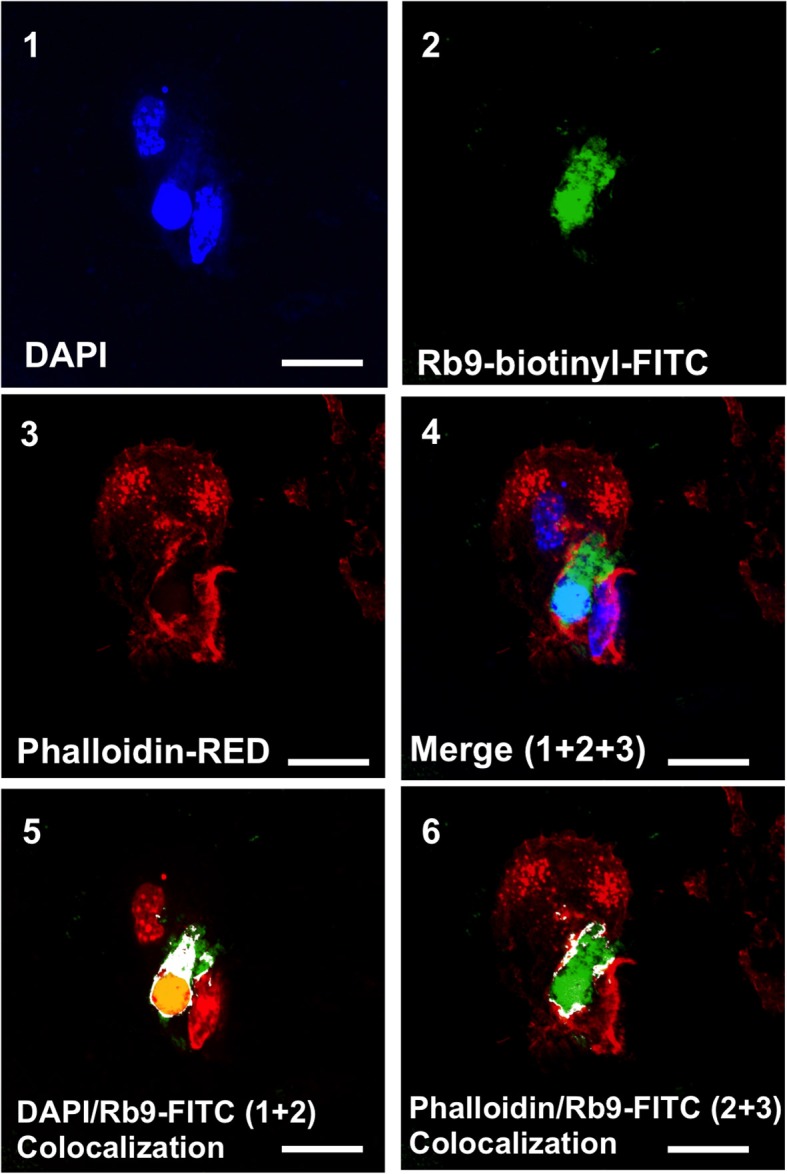
Rb9 interacts with and is internalized by bone marrow derived dendritic cells (BMDCs). Panels are representative confocal images of bmDCs stained with DAPI (blue, panel 1) and phalloidin (red, panel 3) for nucleic acid and filamentous actin staining, respectively. Biotinyl-Rb9 is stained with FITC (green, panel 2). A merge of 1, 2, and 3 can be seen on panel 4. FITC-biotinyl-Rb9 colocalizes with DAPI in a nuclear region (panel 5) and peripherally with phalloidin (panel 6) as shown by white and yellow points/areas in the cellular cytosol on both colocalization panels. Bars = 10 μm.

Since Rb9 peptide requires the immune system participation to display anti-tumor response in a metastatic melanoma setting a direct interaction of the peptide with dendritic cells was looked for as a possible early step in this process. By using fluorescence microscopy we showed that murine bmDCs interact directly with biotinylated Rb9, as revealed with FITC-streptavidin (green staining in Figure 4, panels 2, 4, 5, and 6). Confocal microscopy showed colocalization points of FITC-complex-Rb9 signal and perinuclear regions, DAPI signal, within bmDC (panel 5), and also with actin cytoskeleton stained with red phalloidin (panels 3, 4, and 6). Presumably, after internalization Rb9 could be carried to the nuclear site via actin filaments to participate in a cell-signaling pathway.

### Direct Binding of Rb9 Peptide to MIF and CD74 Proteins

The macrophage migration inhibitory factor (MIF) is synthesized by epithelial and endothelial cells, T lymphocytes, macrophages, and by several tumors, particularly melanoma (35, 36). MIF exerts its oncogenic effects through binding to CD74 receptor, CXCR4 chemokine receptor, and CD44, involved in MIF cell-signaling (37).

**Figure 5:**
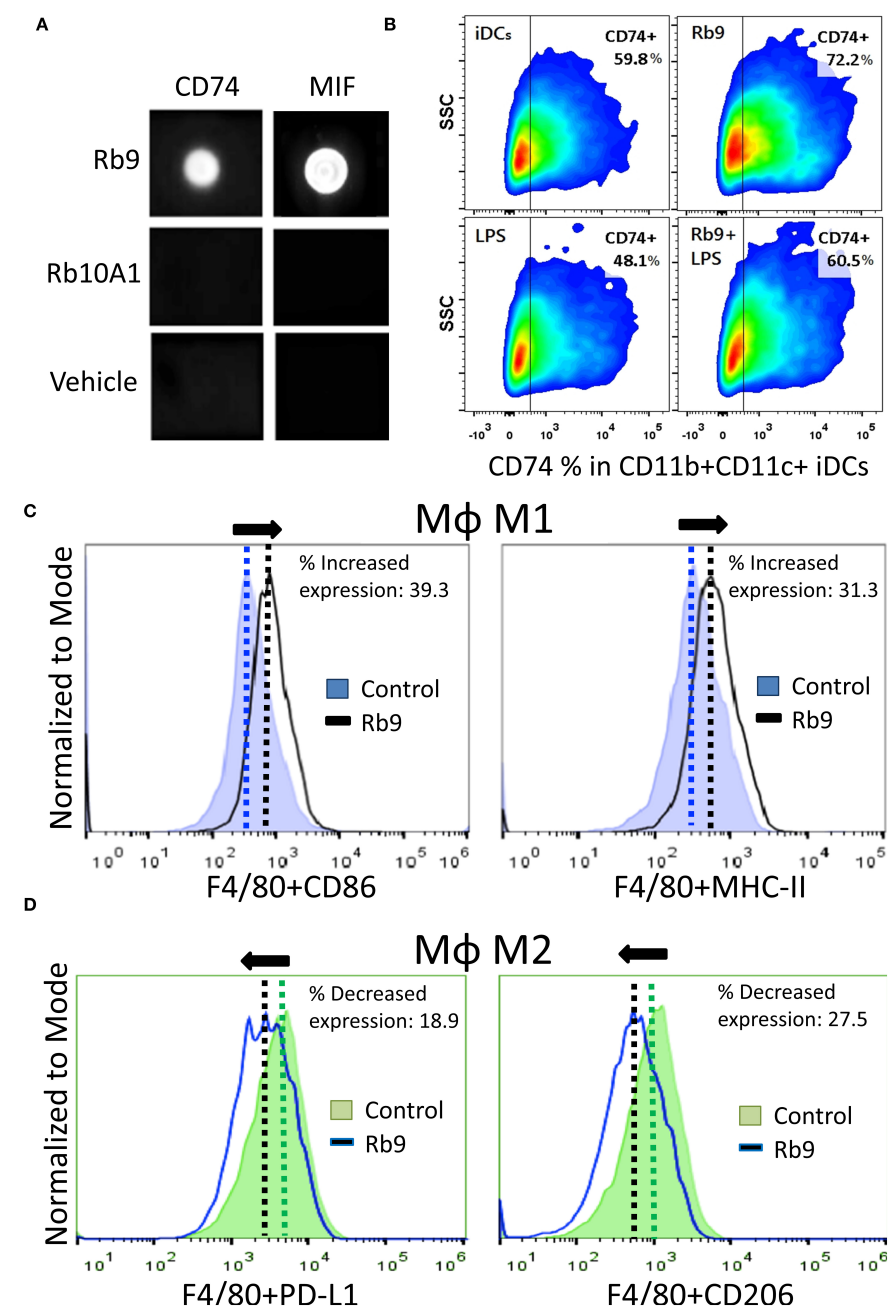
Specific binding of Rb9 peptide to MIF or CD74 recombinant proteins. **(A)** Dot-blotting showing specific interaction of Rb9 peptide with rCD74 and rMIF proteins. No reaction was seen with Rb10A1 peptide or the peptide vehicle (Veh); **(B)** Rb9 increases the expression of CD74 in CD11b+CD11c+ iDCs; **(C)** Increased M1 MΦ marker expression (CD86+ and MHC-II+) in bmMΦs incubated with Rb9 in the presence of melanoma factors (B16F10 conditioned medium, B16.CM); **(D)** Decreased M2 MΦ marker expression (PD-L1+ and CD206+) in bmMΦs incubated with Rb9 in the presence of melanoma factors (B16.CM).

Rb9 peptide binds to MIF and CD74 as shown by dot blotting with the respective recombinant proteins (Figure 5A). A peptide derived from linear Rb10 replacing the N-terminal cysteine by alanine, Rb10A1, Girola et al. (22) was unreactive and also used as a control for *in vitro* experiments. Rb9 also increased CD74 expression in immature dendritic cells (iDCs) expressing CD11b and CD11c, also reversing the negative effect in iDCs of treatment with 200 ng of LPS for 24 h as observed by flow cytometry (Figure 5B). MIF is commonly secreted by B16F10 in cultured melanoma cells, as detected in the conditioned medium (38). Another, previously studied, CDR peptide (C36L1) showed ability to bind to MIF's receptor CD74 and also interfere in the melanoma-secreted factor that regulates macrophage function (38). Based on these results, bone marrow-derived macrophages (bmMΦs) were evaluated in the presence of melanoma factors (B16F10 conditioned medium, B16.CM) with or without Rb9. M1 macrophages expressing CD86 and MHC II showed increased expression upon treatment with Rb9 (B16.CM, Figure 5C). In contrast, decreased expression of M2 macrophages expressing PD-L1 and CD206 was observed in response to Rb9 (Figure 5D).

### Rb9 Interferes With MIF Signaling Pathways in Murine bmDCs

**Figure 6:**
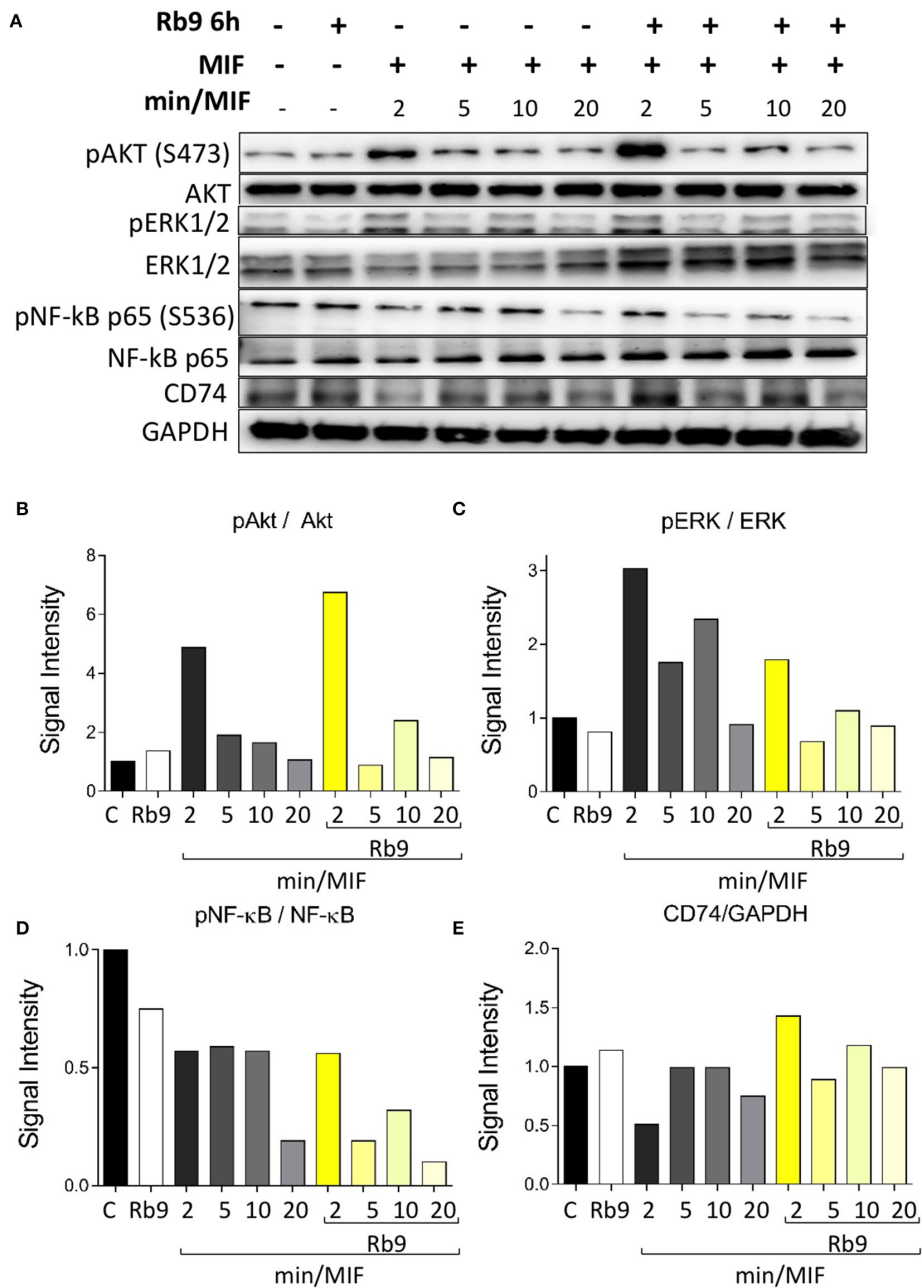
Rb9 pre-incubation modifies the intracellular Akt, ERK, NF-κB signaling pathways and CD74 expression in MIF-stimulated murine bmDCs. **(A)** Western blotting bands of Akt, pAkt (Ser473), ERK1/2, pERK1/2, NF-**κ**B pr65, pNF-**κ**B pr65 (Ser536), CD74, and GAPDH (loading control) from bmDCs pre-incubated for 6 h with/without 200 μM of Rb9 and treated with 1 μg/mL of rMIF for 2, 5, 10, and 20 min; **(B)** Rb9-pretreated cells showed increased signal intensity of pAkt S473 on short incubation with rMIF; **(C)** pERK1/2 in relation to total ERK1/2 decreased in Rb9-pretreated cells in response to rMIF; **(D)** pNF-κB pr65 S536 showed a fast decrease in Rb9-pretreated cells at 5–10 min of rMIF incubation; **(E)** the expression of CD74 increased with Rb9 pre-incubation and combining Rb9 and rMIF.

As Rb9 was shown to interact with bmDCs and, at molecular level, with MIF and CD74 proteins, intracellular signaling pathways were evaluated in DCs in response to rMIF incubation and Rb9 modulatory effects (Figure 6 and Supplementary Figure 4). In Figure 6A, signaling proteins in bmDCs, previously incubated for 6 h with Rb9 at 200 μM, and then treated with 1 μg/mL of rMIF for 2, 5, 10, and 20 min are shown. Cellular extracts at each time-point of rMIF treatment of Rb9-pretreated DCs, were collected and the total and phosphorylated protein levels of Akt, ERK1/2, NF-κB p65, and CD74 were quantified by immunoblotting methods and band densitometry as shown in Figures 6B–E. Overall, Rb9 treatment alone did not significantly change intracellular signaling mediators in bmDC. Nevertheless, when Rb9-preincubated bmDCs were treated with rMIF, Akt phosphorylation at serine 473 (S473) was stimulated after 2 min and less so after 10 min incubation (Figure 6B). In contrast, phosphorylation of ERK1/2 (Figure 6C) in Rb9-bmDCs was significantly reduced by rMIF after 2–10 min incubation, as compared to bmDCs without pretreatment with Rb9. A similar reduction by rMIF in Rb9-bmDCs was observed after 5–10 min incubation, equally compared to bmDCs without Rb9 pre-treatment. For PI3K and IkBα signaling, Rb9 reduced by half the expression of pPI3k, without reversion by rMIF. As to pIkBα little or no difference of rMIF stimulation in bmDCs treated or not with Rb9 was seen (Supplementary Figures 3A–C). Finally, the MIF receptor CD74, showed increased expression when rMIF was added to Rb9-bmDCs for 2 min (Figure 6E) compared to the Rb9-untreated counterpart.

### Therapeutic and Prophylactic Anti-tumor Protection by Adoptive Cell Transfer of Rb9-Stimulated Dendritic Cells

**Figure 7:**
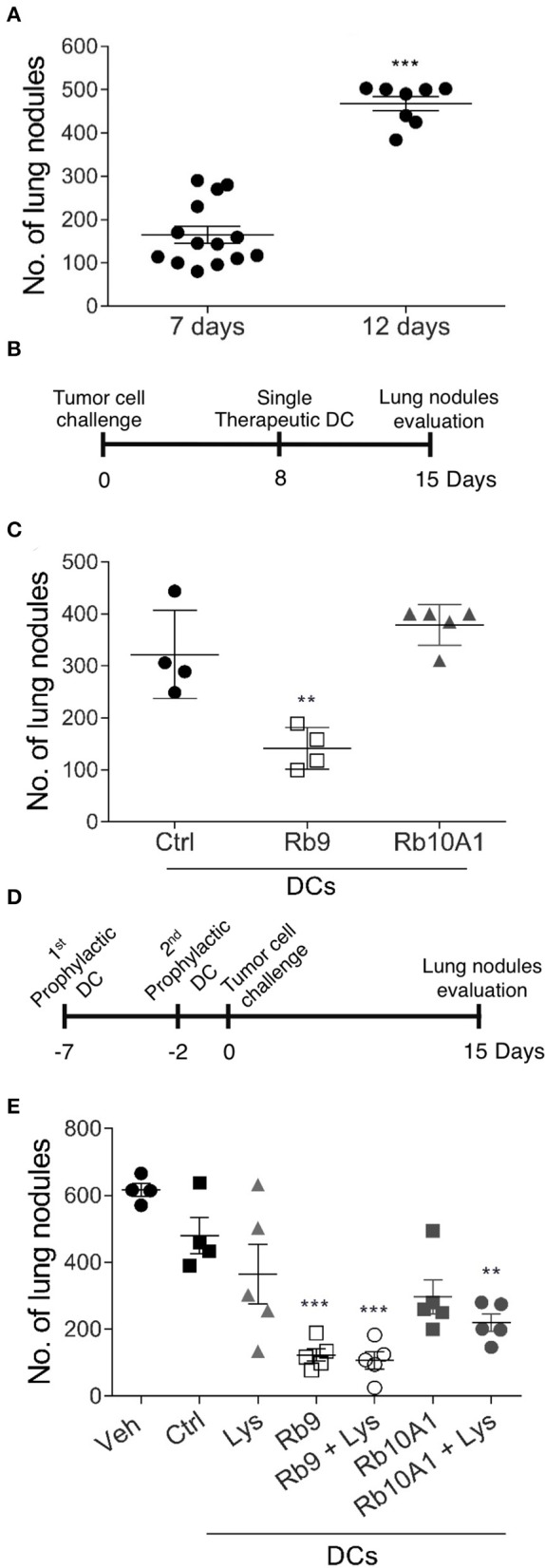
Therapeutic and prophylactic adoptive cell transfer (ACT) using Rb9-stimulated dendritic cells (DCs). **(A)** Syngeneic mice injected i.v. with B16F10-Nex2 melanoma cells show increased number of lung metastatic nodules after 7 and 12 days of tumor cell challenge; **(B)** in a therapeutic protocol Rb9-stimulated bmDCs are injected once after 8 days of tumor cell i.v. challenge; **(C)** metastatic nodules have growth arrested even after 15 days as compared to Rb10A1 control peptide; **(D)** in the prophylactic protocol Rb9 alone or combined with tumor lysate, given *ex-vivo* to bmDCs, are injected twice before tumor cell challenge **(E)** and arrest metastatic growth of melanoma. For this protocol, bmDCs were stimulated with 50 μg/mL of Rb9, Rb10A1 with or without 5 x 10^4^ B16F10-Nex2 cell lysate (Lys) for 24 h previously to s.c. inoculation of mice at 7 and 2 days before melanoma tumor cell challenge. Graphs **(C,E)** show individual values, with means ± SD. ^**^p < 0.01; ^***^p < 0.001 calculated using One-Way ANOVA and Bonferroni correction compared to bmDCs Ctrl groups.

The immune system dependence of Rb9 anti-tumor protection *in vivo* was further explored testing bmDCs treated *ex vivo* with Rb9 and adoptively transferred into tumor-bearing mice (Figure 7). When syngeneic C57Bl/6 mice are i.v. challenged with B16F10-Nex2 cells, a predictable number of metastatic lung nodules in each animal can be represented as a cluster range after 7–8 days and then another after 12–15 days after tumor challenge injection (Figure 7A). Using this standard response, the protective effect of adoptive bmDCs stimulated *ex vivo* with Rb9 or Rb10A1 (negative control) was shown using a therapeutic protocol in which DCs were subcutaneously transferred 8 days after tumor cell challenge (Figures 7B,C). Rb9-DCs suppressed metastatic melanoma progression at 15 days after tumor injection, with the number of metastatic nodules equivalent to that of untreated mice after 8 days of B16F10-Nex2 inoculation (Figure 7A). In a prophylactic protocol, bmDC were previously primed *ex vivo* with tumor cell lysate (Lys) and stimulated with Rb9 or Rb10A1 peptides. Primed cells were injected twice prior to a single B16F10-Nex2 challenge inoculation (Figure 7D). Mice receiving Rb9-stimulated DCs, with or without Lys-priming, were best protected against metastatic melanoma after 15 days of challenge (Figure 7E).

### Rb9 Affects Human moDCs' Surface Phenotype and Enhances Their Ability to Stimulate Allogeneic Lymphocyte Proliferation

**Figure 8:**
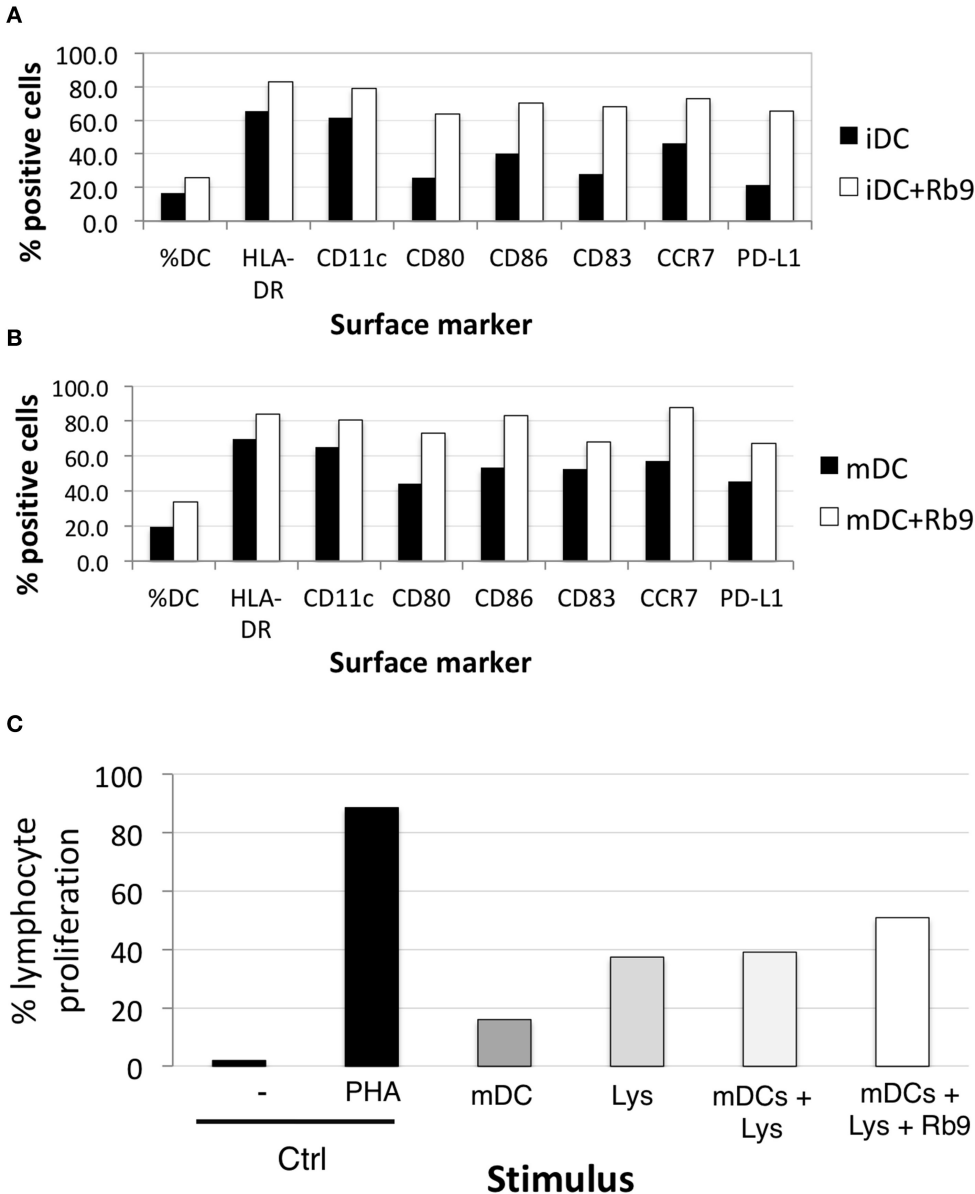
Rb9 affects the phenotype of human monocyte-derived dendritic cells. PBMC from healthy human donor were differentiated into monocyte-dendritic cells and submitted to various treatments; **(A)** shows the increased expression of surface markers (HLA-DR, CD11c, CD80, CD86, CD83, CCR7, and PD-L1) after 48 h-treatment of immature DCs (iDCs) with Rb9 (50 μg/mL); **(B)** a similar effect is shown in TNF-stimulated (mDCs) submitted to Rb9-stimulation; **(C)** PBMC cells from healthy human donor were differentiated into mDCs, submitted to different treatments and used to stimulate CFSE-labeled allogeneic lymphocytes. Enhanced T cell proliferation was observed when mDCs were pulsed with tumor lysate (Lys) and treated with Rb9.

We evaluated the expression and activation of markers in human dendritic cells under Rb9 interference. The capacity of these DCs to stimulate allogeneic lymphocyte proliferation (Figure 8) was investigated with a healthy donor's dendritic cells differentiated *ex vivo* from blood monocytes and incubated with Rb9 alone (iDCs, Figure 8A) or incubated with Rb9 and TNFα (mDCs, Figure 8B). As both figures show, regardless of TNF stimulation, Rb9 treatment increased the expression of HLA-DR, CD11c, CD40, CD80, CD86, and PD-L1 human monocyte-derived DCs (hu-moDCs). Coherently, when hu-moDCs previously pulsed with tumor lysate and treated with Rb9 were co-cultured with allogeneic lymphocytes, the allostimulatory activity was higher than with hu-moDCs alone or combined with tumor lysate-pulsed condition, but without Rb9 treatment (Figure 8C).

**Figure 9:**
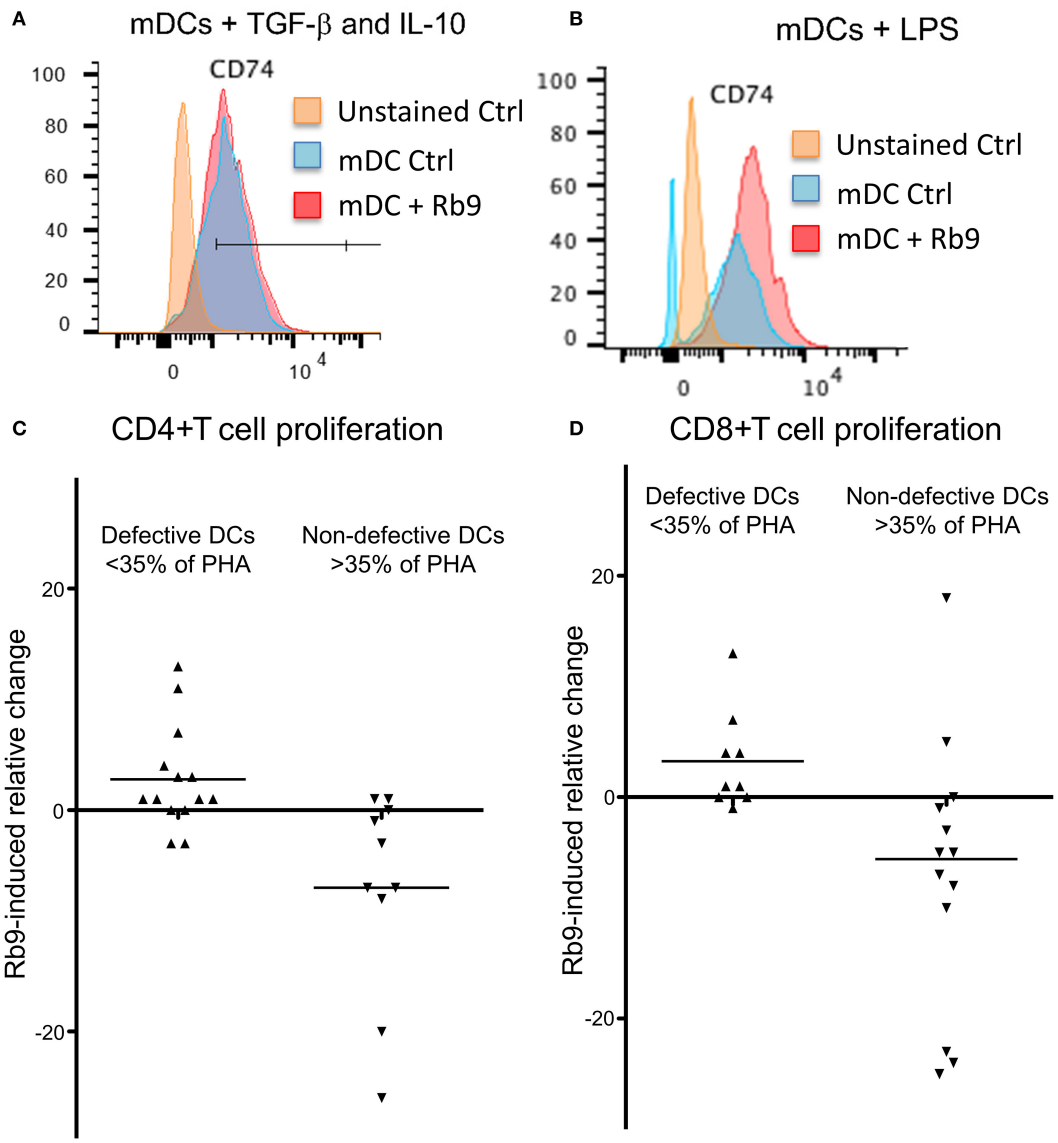
Rb9 treatment of mDCs, from healthy donor and from 22 cancer patients. Ability to stimulate allogeneic lymphocytes' proliferation. Healthy donor' iDCs were stimulated to mDCs with TNF. They were also **(A)** simultaneously treated with TGF-β (10 ng/ml) and IL-10 (1 ng/ml) and further stimulated with Rb9 showing no change in CD74, the MIF receptor; **(B)** treatment with LPS and stimulation with Rb9, caused increased expression of CD74. Allogeneic CD4+ T cell **(C)** and CD8+ T cell **(D)** proliferative responses, were induced by Rb9-treatment of mDC differentiated from 22 cancer patients' PBMC. This depended on the ability of non-treated cancer patients' mDCs to stimulate T cells to proliferate: some had a poor allo-stimulatory activity (<35% the proliferation induced by phytohaemagglutinin, PHA), while others had not this same defective functional phenotype, for both CD4+ and CD8+ T cells. Rb9 treatment increased the lympho-stimulatory proliferation of “defective” mDCs, but decreased the same ability in non-defective mDCs.

Curiously, when hu-moDCs were submitted to stimuli that either over-activated them (LPS) or induced a tolerogenic phenotype (TGF-β + IL-10), Rb9 treatment had opposite effects. After tolerogenic stimuli with TGF-β and IL-10, RB9 treatment overcame the down-regulation of activation markers, increasing the expression of HLA-DR, CD80, CD83, and CD86. In contrast, hu-moDCs earlier hyperstimulated with the LPS treatment after receiving Rb9 showed a down-regulation of activation markers. The significance of these results was evaluated using a X^2^ statistics (Table 1) on the flow cytometer data shown in Supplementary Figure 4. Interestingly, Rb9 treatment increased the expression of the MIF-receptor CD74 in LPS-activated cells, but not in tolerogenic hu-moDCs (Figures 9A,B). CD44 was downregulated in TGF-β + IL-10-treated hu-moDCs, with no alteration on CXCR4 expression (Supplementary Figure 5).

**Table 1:** Significant effects of Rb9 (χ^2^ statistics) on different populations of human neutral (TNF), activated (LPS), or suppressed (IL-10 + TGF-β) dendritic cells (DCs)^a^.

**mDC markers**	**Systems Rb9 and controls**	**Statistics of Rb9 effects**	**Stimuli**
CD11c	LPS	C	
HLA-DR	LPS + Rb9	X^2^ *p* < 0.05	^*^Neg
	TNF	C	
	TNF + Rb9	X^2^ NS	0
	IL-10 + TGF-β	C	
	IL-10/TGF-β + Rb9	X^2^ *p* < 0.01	^**^Pos
CD83	LPS	C	
HLA-DR	LPS + Rb9	X^2^ NS	0
	TNF	C	
	TNF + Rb9	X^2^ NS	0
	IL-10 + TGF-β	C	
	IL-10/TGF-β + Rb9	X^2^ *p* < 0.01	^**^Pos
CD80	LPS	C	
CD86	LPS + Rb9	X^2^ NS	0
	TNF	C	
	TNF + Rb9	X^2^ NS	0
	IL-10 + TGF-β	C	
	IL-10/TGF-β + Rb9	X^2^ *p* < 0.01	^**^Pos

a Results obtained in a flow cytometer (see Supplementary Figure 4). C, control system; X^2^, Chi- squared statistics;

* Neg, significant inhibitory effect;

** *Pos, significant activation; NS, not significant effect*.

Further, hu-moDCs isolated from 22 cancer patients were treated with Rb9 before allostimulation with healthy donor lymphocytes (Figures 9C,D). Since hu-moDCs from cancer patients frequently show deficits in their allostimulatory ability (39, 40), the response in this experiment was evaluated against a positive control: the proliferative response induced by the mitogen, phytohemagglutinin A (PHA). Hu-moDCS from some patients, indeed, showed a “defective” activity (<35% of the response induced by PHA), while others had a “non-defective” activity (more than 35% of the response induced by PHA). Rb9 treatment in defective mDCs was able to slightly increase CD4 and CD8 T cell proliferation (Figures 9C,D) and, in contrast, decreased the response induced by “non-defective” cells.

Overall, these results suggest that Rb9 can modulate human monocyte-derived dendritic cells activity, especially in cancer patient cells, stimulating tolerogenic cells (biased either *in vitro*, by TGF-β with IL-10, or *in vivo* by the presence of cancer) and containing the activation of hyperstimulated cells (by LPS, for example).

## Discussion

Synthetic peptides derived from Complementary Determining Regions (CDRs) of monoclonal antibodies frequently display anti-infective properties and anti-tumor effects (14, 32, 41–43).

Peptide AC1001-H3, derived from V_H_ CDR3 of anti-blood group A mAb showed apoptotic and autophagic effects in murine B16F10-Nex2 melanoma cells (44). The same peptide exerted anti-metastatic activity in C57Bl/6 syngeneic model as well as an immunomodulatory effect in macrophages (45). The PI3K-Akt signaling pathway and the increased expression of TLR-4 induced by TNF-α were characterized in this system.

Hypervariable complementarity determining regions (CDRs) from both light and heavy chains of immunoglobulins are sources of bioactive peptides, acting in many cases, such as V_H_CDR3, as mini-antibodies (41). The immunoglobulin-superfamily (IgSF) carries the greatest number of domains with peptide sequences displaying biological activities including immunomodulatory ones. IgSF proteins make up over 2% of human genes, the largest family in the human genome (46).

As focused on in the present work, Rb9, derived from V_H_CDR3 of RebmAb 200, has a configuration similar to that studied by Morea et al. (47), adding C-terminal amino acids QGC and a C-C disulfide bridge to make it cyclic. A stable structure with numerous H-bonds, internal α-helix, and the disulfide in a hairpin were described in this peptide (22). *In vitro*, Rb9 interacts with HSP90, an adhesion G protein, and surface peroxiredoxin 1, the result being inhibition of melanoma cells migration and invasion (22). As presently shown, Rb9 is protective against metastatic melanoma but the results *in vivo* depend instead on an uncompromised immune system. In fact, dendritic cells (DCs) appear to be involved in the immune response induced by the peptide, since this protective activity of the latter could be reproduced by adoptive transference of DCs, treated *ex vivo* with the peptide, in melanoma-challenged susceptible animals. Therapeutic and prophylactic protocols were effective. With the prophylactic protocol, we observed that pre-treatment of DCs with melanoma lysate did not increase the efficiency compared to Rb9 alone, suggesting that the most important priming occurred *in vivo* after challenge with B16F10 cells, possibly resulting in extensive cell lysis due to NK activity, perforins, and IFN-γ dependent and independent mechanisms (48). Subcutaneous administration of Rb9 seems to be the preferred one, but it seems clear that whatever is the route of inoculation in a tumor-bearing experimental animal, the peptide reacts with local and recruited DCs, modulating their activity in a way that leads to anti-tumor effect and prolonged survival of the host. In the protocols used, depending on the possibility of low supply of tumor antigens, a melanoma cell lysate was used as a primer for cross-presentation by DCs. It is clear, however, that the nature of Rb9 activity is an immune modulatory one. Intraperitoneal administration of Rb9 was also effective in delaying s.c. growth of syngeneic pancreatic and colon cancer cells rather than s.c. B16F10-Nex2 melanoma.

The interaction of biotinyl-Rb9 with DCs was explored using confocal microscopy. Co-localization points were seen with actin-reacting phalloidin and condensed nuclear material, suggesting that the peptide could be carried to the nucleus via F-actin, eventually to mediate a signaling pathway. The discovery that Rb9 binds to rCD74 and rMIF, and the fact that melanoma cells *in vitro* and metastatic tumors secrete MIF (38), prompted us to functionally compare Rb9 with peptide C36L1. This peptide was shown to restore M2 macrophages and DC's immunogenic functions so as to inhibit metastatic tumor growth in lungs. M2 cells characterized by IL-12^lo^IL-23^**lo**^IL-10^**hi**^TGF-β^**hi**^, mediate Th2 responses, immune regulation, and tumor promotion (49). M1 macrophages, with IL-12^hi^IL-23^hi^IL-10^lo^ phenotype, are effector cells in Th1 responses and mediate resistance against tumors. They efficiently produce ROS and NO and inflammatory cytokines, IL-1β, TNF, IL- 6 (50).

Rb9 increased the expression of CD74 in CD11b+CD11c+ dendritic cells and this effect was less intense in iDCs activated by LPS. It was found that a combination of poly(I:C) and LPS with IFNα+γ downregulated the expression of CD74 (51). This effect could be reversed by the immunomodulatory action of Rb9, raising the question of the complex protection mechanism of the peptide against tumors. In fact, CD74 has been shown to negatively regulate DC migration. On the other hand, Rb9 increased M1 markers (CD86 and MHC II) in bmMΦs in the presence of B16F10 conditioned medium (B16.CM) and decreased M2 CD206 marker and PD-L1 in IL-4 polarized M2 bmMΦs also in B16.CM. rMIF signaling in bmDCs was modified by Rb9 pretreatment, particularly increasing the expression of CD74 and decreasing that of pERK and pNF-κB. All these effects pointed to a protective effect of Rb9 in face of the immune suppressive and tumor promoting activities of MIF. This cytokine, abundantly produced by melanoma cells, preferentially stimulates M2-macrophage differentiation. The CD74 receptor is also constitutively expressed on melanoma cells and the MIF-CD74 pathway is correlated with increased PD-L1 expression in cancerous cells (52). Interfering in the MIF-CD74 axis may affect signaling in macrophages and dendritic cells (DCs) that can downregulate immunosuppressive factors and activate cytotoxic T cells (38).

Granzyme B+ and T-CD4+ were examined by IHC in the lung nodules of metastatic melanoma treated with Rb9. Both cytotoxic T lymphocyte (CTLs) and NK cells use the serine protease granzymes as their major death effectors (53, 54). Staining of granzyme B reflected the significant presence of CTLs and NK cells in the tumor nodules, in response to Rb9, and represents the signature of immune effector cells infiltrating the lung tissue (55, 56). In the NK cell population, PD-1 blockade immunotherapy can activate those cells to infiltrate melanoma and lung tumors to elicit anti-tumor responses (57, 58). In the case of NKG2D, increased NKG2D ligand in the tumor cells enhances NK cell cytotoxicity (59). The significant difference in T-CD4+ cells was detected only by flow cytometry in the lung nodules. As to human monocyte-derived dendritic cells (hu-moDC), Rb9 generally increased the expression of maturation markers and other surface molecules, with or without TNF activation of DCs. Rb9 stimulated lymphocyte proliferation associated to mDCs, further confirmed an immune modulatory activity of the peptide.

Hu-moDC represent an effective alternative for naturally occurring DCs, which due to their scarceness are not suitable for use in clinical protocols, but can be replaced by hu-moDC, that can be generated *in vitro* from easily obtainable precursors (60, 61). Dendritic cell-based vaccines still fail to reach the theoretical potential attributed to them (62), probably because DCs within tumors (39) and from circulating precursors in cancer patients (40) are functionally biased and frequently unable to induce effective anti-tumor T lymphocytes. The correction of this bias is an attractive way to develop cancer immunotherapy. Presently, the Rb9 effects were analyzed in mDCs from 22 cancer patients' monocytes. These mDCs can be functionally biased; therefore, two groups were set apart, according to their ability to induce allogeneic T cell proliferation. Patients' mDCs, which induced <35% the response to phytohemagglutinin A (PHA) were considered as “defective” and those that induced a response higher than 35% that of PHA, as “normal.” Rb9 clearly affected the phenotype of the cells from the “defective” mDC group, but had little effect upon “normal” mDCs. Such variation in Rb9 effects was more significant when the ability to induce allogeneic T cell proliferation was focused on. In this sense, Rb9 showed contrasting effects: it enhanced the ability of “defective” mDC to induce T cell proliferation, whereas it inhibited the lymphostimulatory activity of “normal” mDC. Rb9 was not a simple activator of DCs but, actually, a molecule that induced restoration of function in these cells on both directions. To test this hypothesis, hu-moDCs were generated in conditions that lead to the generation of immune response-inducing DCs or to the generation of tolerance-inducing DCs. For the response-inducing DCs, two different stimuli were used, TNF and LPS. TNF is a stimulus that induces a “mild” activation of the cells, thus resembling a close-to-homeostasis condition, while LPS is a stronger stimulus, signaling a more disturbed environment. For the tolerance-inducing DCs, TGF-beta and IL-10 were used (63). When Rb9 was added to these three different hu-moDCs, all in the presence of GM-CSF and IL-4 both at 50 ng/mL, the immune modulatory effects were evident. While Rb9 little affected the phenotype of TNF-stimulated hu-moDCs, it induced a decreased expression of maturation markers in LPS-stimulated hu-moDCs and an increase in the same markers on [TGF-β+IL-10]-stimulated hu-moDCs. A similar contrasting effect of Rb9 was noticed when the frequency of mature, HLA-DR+CD83+cells was determined, and when cells double positive for the co-stimulatory molecules, CD80 and CD86 were evaluated.

The effects of Rb9 peptide in nature and particularly those that play a role in the defense against tumors are complex, involving a great number of interacting molecules, strictly dependent on the experimental system set up for their investigation. Starting from the peptide protective activity against metastatic melanoma in susceptible mice, we evolved to immunological responses that have a counterpart in human immune mechanisms including cells from human cancers. Some interactions were found relevant to suggest predominant mechanisms of action *in vivo*, quite different from those previously described *in vitro* for the same melanoma cell line, acting directly on the cultured tumor cells without participation of the immune system (22). The anti-tumor protective effect *in vivo* by Rb9 involved mainly dendritic cells, T cell effector lymphocytes, cytokines, and several regulatory mechanisms of which the MIF-CD74 interaction appears to be most relevant. In fact, Rb9 binds to CD74 and to MIF, increases the expression of CD74, modify both the macrophage phenotype, increasing type M1, and bmDC signaling, decreasing pERK, pNF-kB, and pPI3K (alone or with MIF). Rb9 in the murine system also increases IFN-γ, which upregulates CD74, and decreases IL-6 (64) and TGF-β. In human moDCs Rb9 increases CD74 when activated by LPS but not when treated with TGF-β + IL-10. In the latter condition Rb9 decreased the expression of CD44 but not on LPS-activated mo-DCs (Supplementary Figure 5). Recently, MIF-CD74 interaction was identified as a regulator of PD-L1 expression, being therefore a target for melanoma treatment (52). In our laboratory, the subcutaneous immunization with shRNA-SOCS1-transduced viable B16F10-Nex2 tumor cells, which inhibited the expression of PD-L1 rendered significant protection against melanoma in the syngeneic model used (65). Whereas, the most aggressive WT B16F10 strain highly expressed PD-L1, the SOCS1 silenced variant, which lacked PD-L1, significantly lost its virulence suggesting a possible cross-interaction. The effects described for Rb9 and the protection against metastatic melanoma may suggest a potential for this peptide to be associated to modern cancer immunotherapeutic procedures.

## Data Availability Statement

The raw data supporting the conclusions of this article will be made available by the authors, without undue reservation, to any qualified researcher.

## Ethics Statement

The studies involving human participants were reviewed and approved by Research Ethical Committee (CEP, Brazil) from the Faculty of Medicine, São Paulo University (FMUSP, Brazil). The protocol was approved ref. number 457.616 on Nov 13, 2013. The patients/participants provided their written informed consent to participate in this study. The animal study was reviewed and approved by National Council for the Control of Animal Experimentation (CONCEA, Brazil) and approved by the Ethics Committee of Federal University of São Paulo, registered with the number CEUA 3521121217.

## Author Contributions

LT, JB, FM, NG, VM, and PB-S conceived and designed the experiments. FM, VM, NG, PB-S, CF, and RA performed the experiments. LT, FM, and JB organized figures and wrote the manuscript. AM and the above authors helped with analysis and interpretation of results and approved the manuscript.

### Conflict of Interest

LT and JB are scientific advisers for Recepta Bio. FM, NG, VM, and PB-S have been fellows via ProUniemp association in a FAP-UNIFESP Foundation and Recepta Bio sponsored project. AM was an R&D analyst for Recepta Bio. The remaining authors declare that the research was conducted in the absence of any commercial or financial relationships that could be construed as a potential conflict of interest.

## Supplementary Material

Supplementary Figure 1 Rb9 decreased both colorectal and pancreatic syngeneic s.c. grafted tumors in mice. **(A)** Tumor volume of CT26 cell syngeneic colorectal cancer was measured in i.p. Rb9-treated mice with 300 μg/dose for five alternate days, starting on the first day after tumor cell challenge. The values are means ± SEM and ^***^*p* < 0.001 calculated using a ratio paired Student's *t-*test compared to Vehicle (Veh) treatment; **(B)** Tumor volume of Panc02 cell syngeneic pancreatic cancer was measured in i.p. Rb9-treated mice with 300 μg/dose for 5 alternate days, starting on the first day after tumor cell challenge. Graph represent means ± SD and ^***^*p* < 0.001 calculated using a ratio paired Student's *t*-test compared to Veh treatment. **(C)** Survival curve of mice from previous experiment **(B)**, Panc02 SC tumor model. ^*^*p* < 0.01 calculated using Log-rank (Mantel-Cox) test.

Supplementary Figure 2 IL-12, TNF, IL-10, and IL-6 secretion in splenocytes from Rb9-treated mice. **(A)** Splenocytes were collected from 17-day tumor-cell challenged mice, treated with i.p Rb9 or Rb10A1 for five alternate days after melanoma cells inoculation. The splenocyte cell culture supernatants were used to measure cytokine secretion after 72 h stimulus with B16F10-Nex2 lysate **(A–D)**. All panels represent means ± SD of triplicate experiments quantified in ELISA assays using cytokine controls.

Supplementary Figure 3 Effects of Rb9 and MIF treatment on PI3K and IkBα signaling pathways in bmDCs. **(A)** Panels showing Western blotting bands of PI3K p85, pPI3K pr85 (Tyr458), and IkBα, pIkBα (Ser32) from bmDCs, after preincubation or not with 200 μM Rb9 for 6 h, and treated with 1 μg/mL of rMIF for 2, 5, 10, and 20 min; **(B)** Signal intensity of pPI3K p85 T458 showed half decrease in all samples treated with Rb9 or rMIF; **(C)** Signal intensity of pIkBα showed a slight decrease in Rb9-pretreated bmDCs in response to rMIF.

Supplementary Figure 4 Rb9 treatment of different mDC populations. iDCs obtained from human donor PBMC were stimulated to mDCs with TNF. They were also treated either with TGF-β (10 ng/ml) and IL-10 (1 ng/ml) to raise suppressed DCs or with LPS for activated DCs. Control populations examined in a cytometer expressed DCs gated for: **(A)** CD11c/HLA-DR; **(B)** CD83/HLA-DR; and **(C)** CD80/CD86. These three DC populations were further stimulated with Rb9 and the differential response compared to controls treated with TNF; (TNF) + TGF-β/IL-10 or (TNF) + LPS for significance using **X**^**2**^ statistics, as shown in Table 1.

Supplementary Figure 5 CD44 and CXCR4 expression in human mDCs induced by different treatments. PBMC from healthy human donors were differentiated into monocyte-derived dendritic cells, maturated with LPS did not respond to Rb9 **(A)**; with TNF and TGF-β and IL-10 stimulation, Rb9 treatment reduced CD44 but not CXCR4 expression **(B)**.
