# Supplementary figures and images for: Immunomodulatory Protective Effects of Rb9 Cyclic-Peptide in a Metastatic Melanoma Setting and the Involvement of Dendritic Cells
Source: Front Immunol. 2020 Jan 15;10:3122. doi: 10.3389/fimmu.2019.03122 (PMC6974543; doi:10.3389/fimmu.2019.03122)

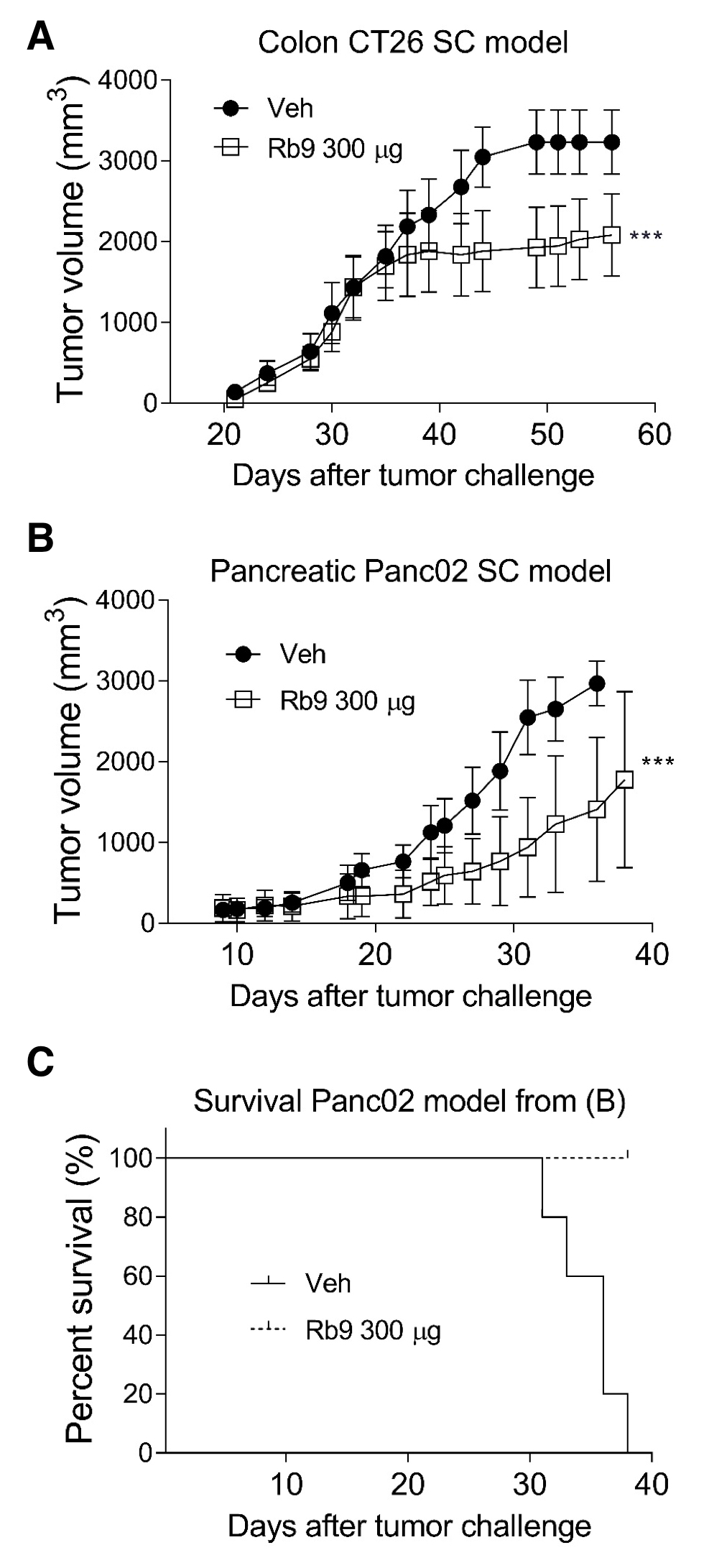

Supplement: Supplementary Figure 1 — Rb9 decreased both colorectal and pancreatic syngeneic s.c. grafted tumors in mice. (A) Tumor volume of CT26 cell syngeneic colorectal cancer was measured in i.p. Rb9-treated mice with 300 μg/dose for five alternate days, starting on the first day after tumor cell challenge. The values are means ± SEM and ***p < 0.001 calculated using a ratio paired Student's t-test compared to Vehicle (Veh) treatment; (B) Tumor volume of Panc02 cell syngeneic pancreatic cancer was measured in i.p. Rb9-treated mice with 300 μg/dose for 5 alternate days, starting on the first day after tumor cell challenge. Graph represent means ± SD and ***p < 0.001 calculated using a ratio paired Student's t-test compared to Veh treatment. (C) Survival curve of mice from previous experiment (B), Panc02 SC tumor model. *p < 0.01 calculated using Log-rank (Mantel-Cox) test. [file Image_1.TIF]

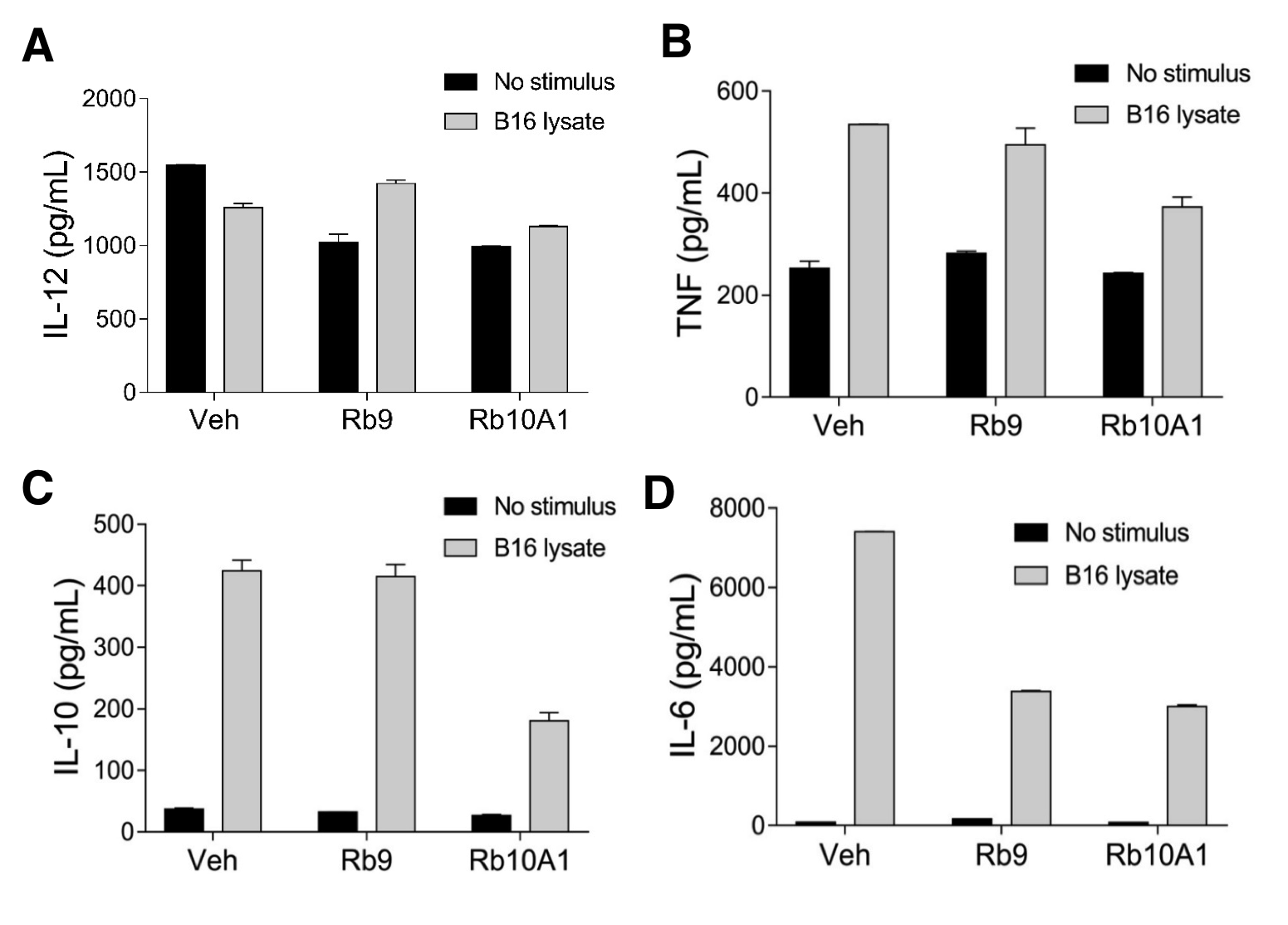

Supplement: Supplementary Figure 2 — IL-12, TNF, IL-10, and IL-6 secretion in splenocytes from Rb9-treated mice. (A) Splenocytes were collected from 17-day tumor-cell challenged mice, treated with i.p Rb9 or Rb10A1 for five alternate days after melanoma cells inoculation. The splenocyte cell culture supernatants were used to measure cytokine secretion after 72 h stimulus with B16F10-Nex2 lysate (A–D). All panels represent means ± SD of triplicate experiments quantified in ELISA assays using cytokine controls. [file Image_2.TIF]

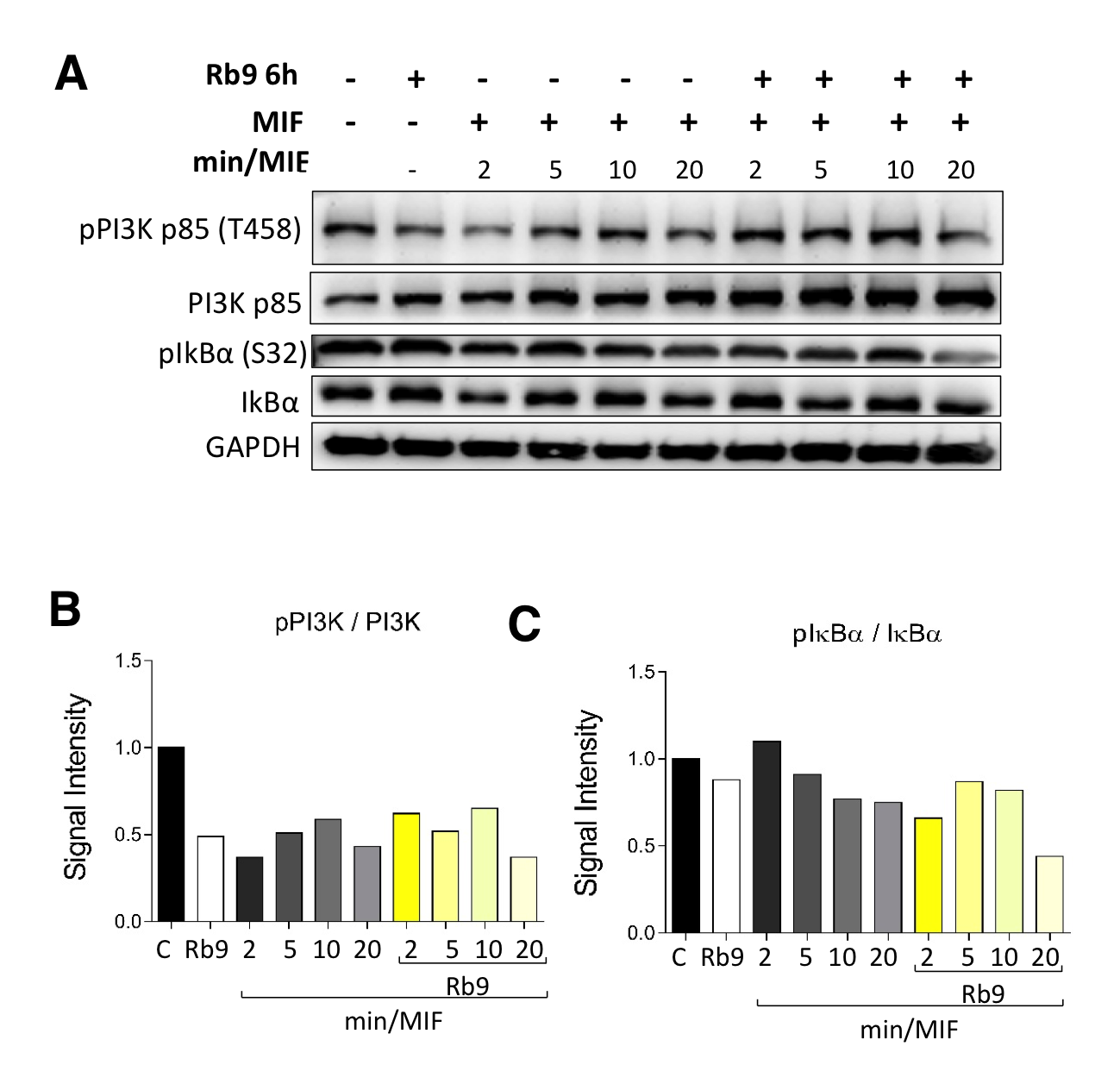

Supplement: Supplementary Figure 3 — Effects of Rb9 and MIF treatment on PI3K and IkBα signaling pathways in bmDCs. (A) Panels showing Western blotting bands of PI3K p85, pPI3K pr85 (Tyr458), and IkBα, pIkBα (Ser32) from bmDCs, after preincubation or not with 200 μM Rb9 for 6 h, and treated with 1 μg/mL of rMIF for 2, 5, 10, and 20 min; (B) Signal intensity of pPI3K p85 T458 showed half decrease in all samples treated with Rb9 or rMIF; (C) Signal intensity of pIkBα showed a slight decrease in Rb9-pretreated bmDCs in response to rMIF. [file Image_3.TIF]

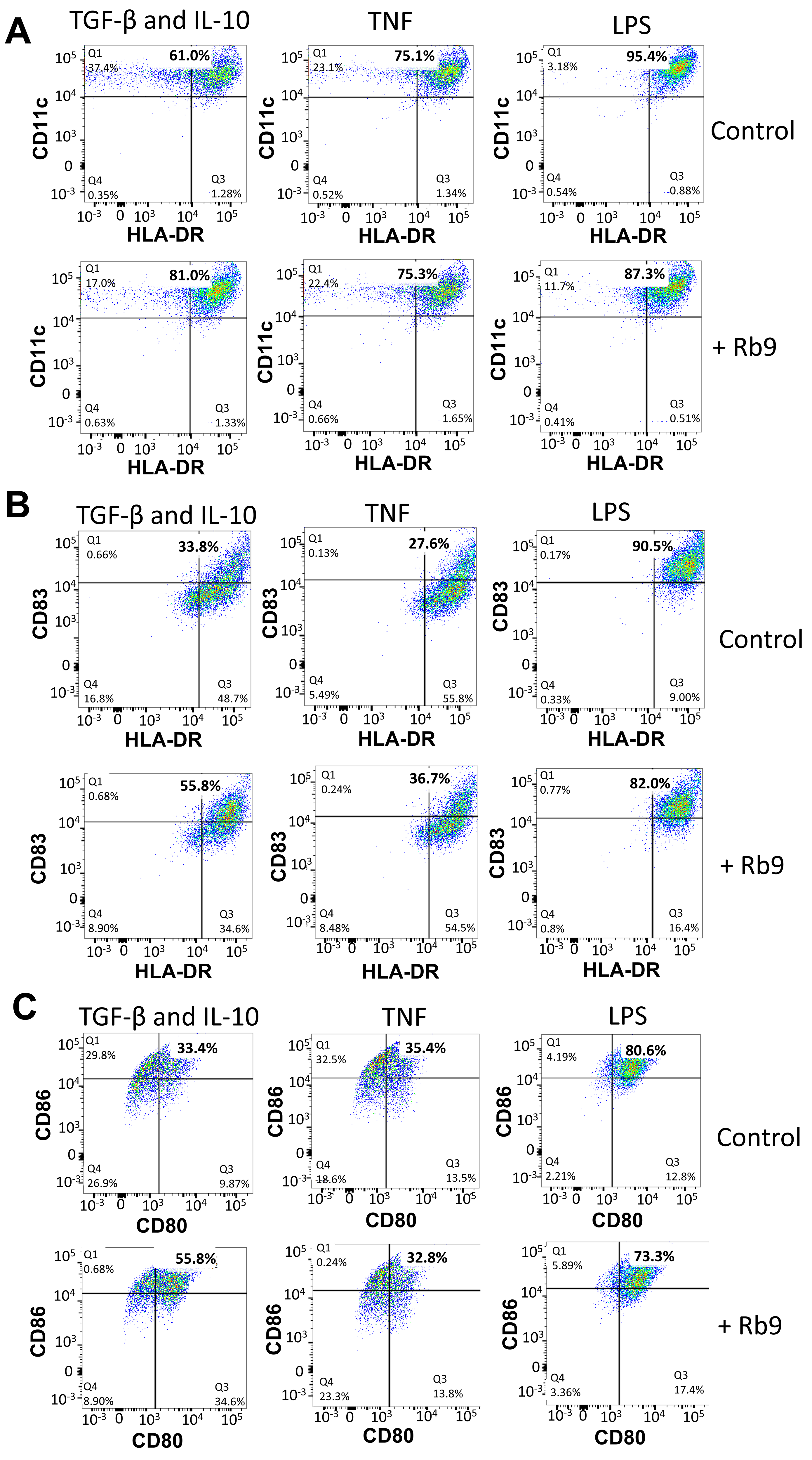

Supplement: Supplementary Figure 4 — Rb9 treatment of different mDC populations. iDCs obtained from human donor PBMC were stimulated to mDCs with TNF. They were also treated either with TGF-β (10 ng/ml) and IL-10 (1 ng/ml) to raise suppressed DCs or with LPS for activated DCs. Control populations examined in a cytometer expressed DCs gated for: (A) CD11c/HLA-DR; (B) CD83/HLA-DR; and (C) CD80/CD86. These three DC populations were further stimulated with Rb9 and the differential response compared to controls treated with TNF; (TNF) + TGF-β/IL-10 or (TNF) + LPS for significance using X2 statistics, as shown in Table 1. [file Image_4.TIF]

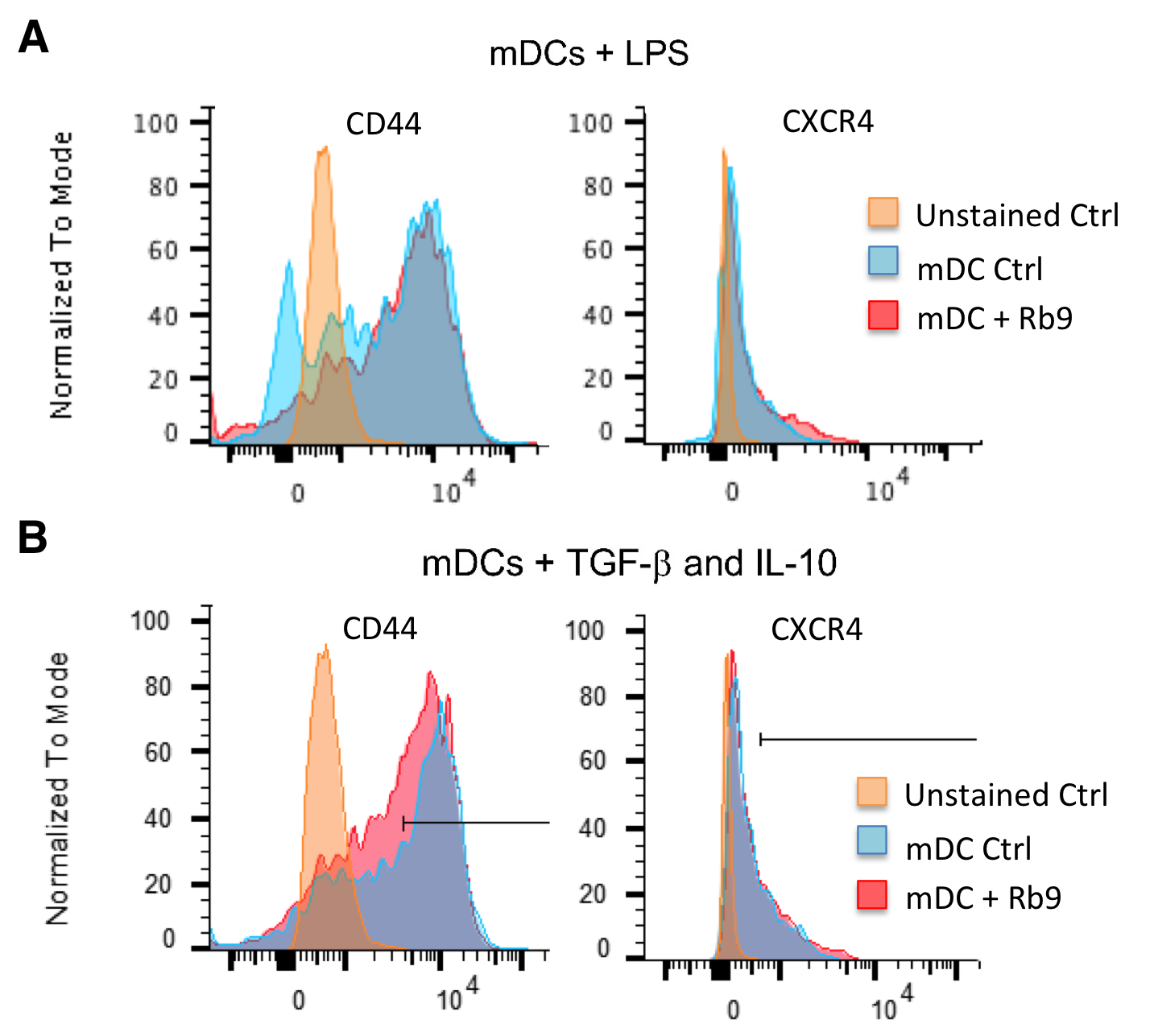

Supplement: Supplementary Figure 5 — CD44 and CXCR4 expression in human mDCs induced by different treatments. PBMC from healthy human donors were differentiated into monocyte-derived dendritic cells, maturated with LPS did not respond to Rb9 (A); with TNF and TGF-β and IL-10 stimulation, Rb9 treatment reduced CD44 but not CXCR4 expression (B). [file Image_5.TIF]
